# Prevalence, determinants, intervention strategies and current gaps in addressing childhood malnutrition in Vietnam: a systematic review

**DOI:** 10.1186/s12889-024-18419-8

**Published:** 2024-04-04

**Authors:** Charlotte Mondon, Pui Yee Tan, Chong Ling Chan, Thuy Nga Tran, Yun Yun Gong

**Affiliations:** 1 School of Food Science and Nutrition, Faculty of Environment, University of Leeds, Leeds, LS2 9JT UK; 2 Department of Micronutrient, National Institution of Nutrition, 48B Tang Ba Ho, Hai Ba Trung District, Ha Noi, Vietnam

**Keywords:** Undernutrition, Overnutrition, Micronutrient deficiencies, Prevalence and determinants, Interventions, Children and adolescents, Vietnam

## Abstract

**Background:**

Childhood malnutrition in all forms is a major public health issue worldwide. This review systematically examined the prevalence and determinants and identify the potential interventions and current gap in addressing malnutrition including undernutrition, overnutrition and micronutrient deficiencies (MNDs) in Vietnamese children aged 0–18 years old.

**Methods:**

Embase, Scopus, PubMed, and Web of Science were systematically searched through June 2022 to identify relevant articles published within the past 25 years. Study selection and data extraction were performed by one reviewer and checked for accuracy by the other two reviewers in accordance with PRISMA guideline. Risk of publication bias was assessed using American Dietetic Association Quality Criteria Checklist.

**Results:**

Seventy-two studies that met the inclusion criteria were included. Undernutrition has decreased over time but still 22.4%, 5.2% and 12.2% of children under 5 were stunted, wasted and underweight, respectively. Anaemia, iron, zinc, and vitamin D deficiencies were the more common forms of MNDs, the prevalence varied by age, region, and socioeconomic group. Population-based surveys reported that 11% and 48% of children aged 0–11 years old were iron and vitamin D deficient, respectively. Zinc deficiency affected almost one-quarter of the children and adolescents. Retinol deficiency was of less concern (< 20%). However, more evidence on MNDs prevalence is needed. Overweight and obesity is now on the rise, affecting one-third of school-aged children. The key determinants of undernutrition included living in rural areas, children with low birth weight, and poor socio-economic status, whereas living in urban and affluent areas, having an inactive lifestyle and being a boy were associated with increased risk of overweight and obesity. Nutrition specific intervention studies including supplementation and food fortification consistently showed improvements in anthropometric indices and micronutrient biomarkers. National nutrition-sensitive programmes also provided nutritional benefits for children’s growth and eating behaviours, but there is a lack of data on childhood obesity.

**Conclusion:**

This finding highlights the need for effective double duty actions to simultaneously address different forms of childhood malnutrition in Vietnam. However, evidence on the potential intervention strategies, especially on MNDs and overnutrition are still limited to inform policy decision, thus future research is warranted.

**Supplementary Information:**

The online version contains supplementary material available at 10.1186/s12889-024-18419-8.

## Introduction

Vietnam is a country in Southeast Asia (SEA), with an estimated population of 96 million. Since 1986, the government has introduced economic reform, aiming to boost the country’s economic development [1]. Vietnam now has become one of the fastest growing gross domestic products worldwide [2], which resulted in substantial decrease in both poverty and undernutrition in the population. However, inequalities have arisen in social, health, and wealth, whilst overnutrition has become an increasing burden [3]. Malnutrition can lead to serious health consequences and increases the risk of childhood mortality and morbidity. Undernutrition affects every system in the body, which results in altered immune function, increased susceptibility to illnesses and the complications include increased respiratory tract infections, impaired cardiac function, and reduced long-term cognitive development [4]. The health consequences that arise from MNDs vary widely depending on the types and magnitudes of deficiency of such micronutrients, but can include xerophthalmia (dry eyes, night blindness), anaemia (fatigue and heart palpitations), and goitre (swelling of the neck). Overnutrition is associated with increased risk of diet-related non-communicable diseases such as cardiovascular disease, cancers, strokes, type 2 diabetes, and polycystic ovarian syndrome [5].

Thi et al. 2015 proposed that the nutrition transition in Vietnam began in the late 1990’s [6] due to rapid economic development, urbanisation, globalisation via free trade and investment, alongside changes in food system, promoted consumption of energy-dense, nutrient-poor foods and sedentary behaviours among children [7]. This shift has led to the development of the double or triple burden of malnutrition, with overweight and obesity becoming more prevalent, whilst undernutrition persists [8, 9]. The latest General Nutrition Survey in 2019 reported that 19.6% and 16.8% of children aged < 5 and 5–19 years old were stunted, respectively, whereas 19% of children aged 5–19 years old were overweight [10]. Determinants found to be associated with child malnutrition can be classified as distal (socioeconomic), intermediate (environmental or maternal) or proximal factors (individual) [11]. Distal factors include the region of living, ethnicity, mother’s education and occupation, and household income. Intermediate factors were split into two types: environment such as household size, structure of house, latrine type, and water source, and maternal variables such as mother’s age at birth, mother’s BMI, and number of children. Proximal factors include quality of breastfeeding (duration and initiation time), birth weight, and health status.

Tackling malnutrition is one of the United Nations Sustainable Development Goals (SDGs), specifically ‘Goal 2 – Zero Hunger’, needing joint efforts from multiple sectors and disciplines. This requires achieving food security, improving diet and nutrition, and promoting sustainable agriculture and consumption. In general, there are two different types of nutrition interventions: nutrition specific and nutrition sensitive [12]. Nutrition specific interventions aim to address any immediate determinants of malnutrition, and these include supplementation and food fortification, which mostly focus on iron, zinc, folic acid or multiple micronutrients, as well as supporting exclusive breastfeeding, and diet diversification [12]. In addition, nutrition sensitive interventions aim to address the underlying root-causes of malnutrition, such as improving food security through agriculture support, nutritional education, safe drinking-water, sanitation, hygiene and improving healthcare access [13].

Given the severity of the malnutrition situation, the key drivers in Vietnamese children have rarely been extensively and systematically reviewed, leading to an evidence gap on the extent and burden of malnutrition and its potential strategies. Therefore, this study aimed to systematically review the current evidence on the prevalence, determinants and intervention strategies of malnutrition including undernutrition (stunting, wasting and underweight), over-nutrition (overweight and obesity) and micronutrient status, particularly that of anaemia, iron, zinc, vitamin A, D, and folate, in Vietnamese children aged 0–18 years old.

## Methodology

This systematic review was conducted by three independent reviewers in accordance with the preferred reporting items for systematic reviews and meta-analyses (PRISMA) guidelines.

### Inclusion and exclusion criteria

PI(E)CO (Participant, Intervention/ Exposure, Comparison, and Outcome) framework [14] was used to guide the development of the inclusion and exclusion criteria of this systematic review, and the details are shown in Table 1. All observational studies (cross-sectional and longitudinal) and human experimental studies (randomised, or non-randomised, and controlled, or non-controlled trials) reporting the prevalence, determinants, and interventions (including both nutrition sensitive and specific programmes) of malnutrition in Vietnamese children aged 0–18 years were included.

**Table 1:** PI(E)CO criteria for inclusion and exclusion of studies

Criteria	Inclusion	Exclusion
Population (P)	Vietnamese infants, young children, and adolescents aged 0–18 years old	Those who have been diagnosed with any chronic or congenital diseases that potentially affect the child’s nutritional status
Exposure (E) and intervention (I)	Any studies that reported the prevalence, determinants of childhood malnutrition, and interventions (including both nutrition specific and sensitive programmes) aiming to address childhood malnutrition	Studies assessing the genetic effects on malnutrition indicators; interventions targeted at mothers or pregnant women with no child outcomes; interventions that only reported the protocol with no outcomes
Comparison (C)	Those who were exposed to the risk factors or interventions related to malnutrition	Not applicable
Outcome (O)	Malnutrition indicators including undernutrition (stunting, wasting, underweight, and thinness), overnutrition (overweight and obesity) and micronutrient deficiencies, assessed by blood biomarkers such as iron (or ferritin, transferrin), vitamin A (or retinol), vitamin B9 (or folate/folic acid), anaemia (haemoglobin (Hb)), zinc, and iodine	Studies that do not assess malnutrition indicators as the primary outcomes or focused on the consequences of malnutrition

Malnutrition indicators included undernutrition which was defined according to the World Health Organisation (WHO) guideline such as stunting (height-for-age Z-score (HAZ) or length -for-age Z-score (LAZ)), wasting (weight-for-height Z-score (WHZ)), thinness (body mass index-for-age Z-score (BAZ)), underweight (weight-for-age Z-score (WAZ) or weight-for-length Z-score (WLZ)), and mid upper arm circumference (MUAC)) [4, 15]. Indicators for overnutrition included overweight and obesity which defined by both WHO and International Obesity Task Force (IOTF), body mass index (BMI), and waist and hip circumferences [16]. MNDs particularly focusing on anaemia, iron, zinc, and vitamins A, D, and B9 (folate or folic acid). Only studies that assessed MNDs using biomarkers were included, whereas those that assessed using dietary intake were excluded.

Only studies with access to full text, reported in English and within the past 25 years were included. This time frame has been selected based on Thi et al. 2015 who reported that the nutrition transition in Vietnam began in the late 1990’s to capture the emergence of the double or triple burden of malnutrition [6]. Studies identified from the search were checked and confirmed with local experts at the National Institute of Nutrition, Vietnam to ensure all or at least most of the relevant studies in this region have been captured. Communication, case reports, letters, editorial matters, conference abstracts and reviews were not included.

### Search strategy

The literature search was performed on 29^th^ June 2022 using four different databases including Embase, Scopus, PubMed, and Web of Science. Details of the search strategies for each database are reported in Table S1.

### Study selection

Studies were exported from each database into EndNote (Endnote X7.7.1, Thomson Reuters 2016) and were screened by three independent reviewers using the inclusion and exclusion criteria. When records were identified from all four databases, the duplicates were removed. During the first stage of screening, all studies were filtered by title and abstract, and those which did not meet the inclusion and exclusion criteria were excluded. The full texts of the remaining records were accessed and screened using the same eligibility criteria. The final decision regarding the eligibility of articles was made by agreement between all reviewers. Disagreement between reviewers were resolved by discussion and by other reviewers when necessary.

## Data extraction

Where available, the following information was extracted: author and year of publication, study design and duration, participants’ characteristics including inclusion criteria, age and sample size, outcome measures (anthropometric parameters and micronutrients related blood biomarkers), intervention approaches (where appropriate), and key findings. In the case of missing data or unclear pieces of information, it was considered that the authors did not report such variables. Any disagreement between the three reviewers was resolved by discussion to reach a consensus, and by involving other authors when necessary.

### Data synthesis

The quantitative data from the studies included in this review were analysed. Data were extracted and tabulated manually into a pre-prepared excel spreadsheet based on three different topics of interest: the prevalence, determinants and the interventions that primarily aimed to address childhood malnutrition in Vietnam. Determinants included those factors found to be negatively or positively associated with different forms of malnutrition; the effect size of such associations (e.g., odds ratios (OR) or β-coefficient) were extracted and reported in the tables, and if it was not shown, the p-value was reported. With respect to interventions, the studies were further stratified into nutrition sensitive or nutrition specific interventions [17]. Definition of nutrition specific and sensitive interventions were as mentioned in the introduction section. The details of the interventions including the duration, sample characteristics, intervention approaches, control group (where applicable), and the key findings assessed by the post-intervention changes in child anthropometric indices, or micronutrients biomarkers were reported.

### Quality criteria checklist for risk of bias

The risk of bias was assessed for each study using a Quality Criteria Checklist from the Academy of Nutrition and Dietetics [18]. This includes questions on [1] research question clarity, [2] selection bias, [3] study group comparability, (4) withdrawal description, (5) blinding, (6) interventions, (7) study procedure description, (8) appropriate statistical analysis, (9) supported conclusions and limitations, and (10) sponsorship or funding bias. Depending on the answers to these questions, each study was assigned a negative, neutral, or positive rating. To be rated positive, five of the questions must be answered yes, including questions 2, 3, 6 and 7, and one other yes. The risk of bias was assessed by one reviewer and cross-checked by the other two reviewers. The results were summarised in a horizontal stacked bar graph, Fig. 2, showing the percentage of studies which had a low, medium, or high risk of bias for each question.

## Results

**Fig. 1:**
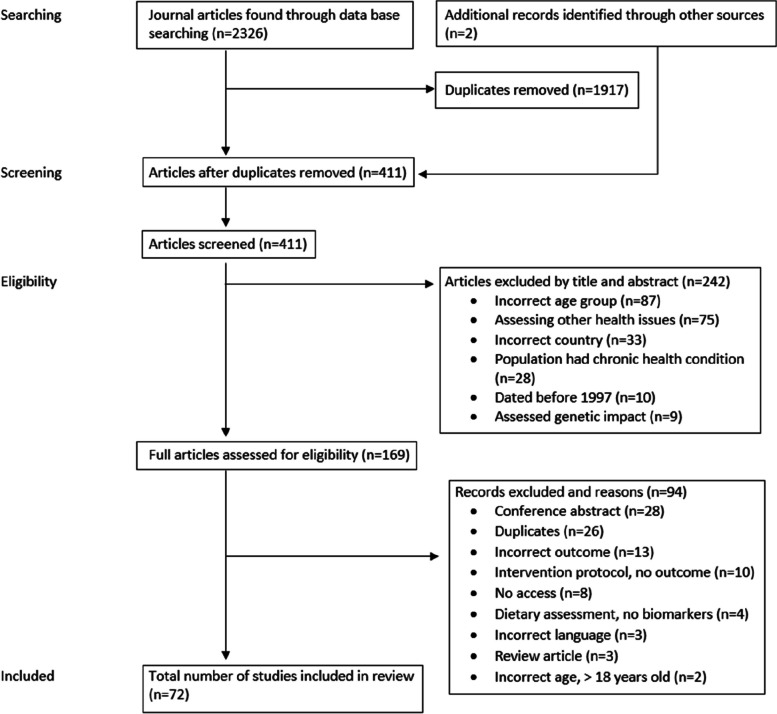
PRISMA flow diagram of identification and selection of studies

As shown in Fig. 1, the initial database search strategy identified a total of 2,326 records. This included 722 from Embase, 620 from PubMed, 571 from Scopus and 413 from Web of Science. Two additional records were identified from the reference list of records from the original search. After removing the duplicates, 411 records were screened by title and abstract to check for eligibility based on the inclusion and exclusion criteria. Of those, 242 articles were removed, and 169 full-text articles were assessed for eligibility. A final total of 72 articles which met the inclusion criteria were then included in this review.

### Risk of bias assessment

**Fig. 2:**
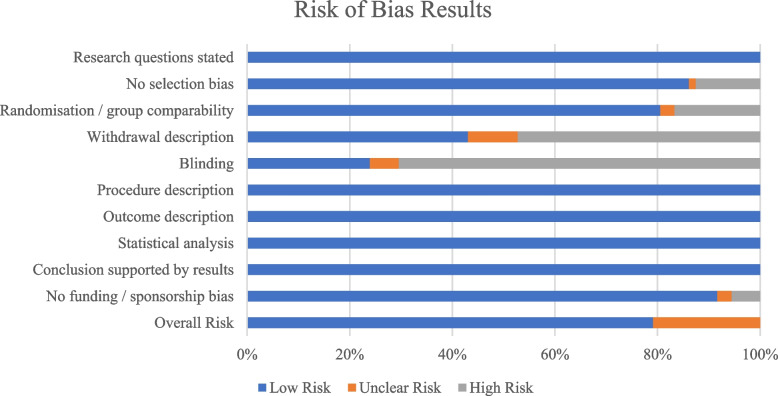
Quality rating of included studies using the Quality Criteria Checklist from Academy of Nutrition and Dietetics

The results for the risk of bias assessment are reported in Fig. 2. 79% of the records were classified as low risk of bias and 21% as unclear risk of bias. The common reasons for lower quality rating were typically the lack of blinding (69% of the records), lack of a withdrawal description (47%) and lack of randomisation or group comparability (17%). All studies stated that the research question, procedure, and the outcome measures have been sufficiently described.

### General characteristics of the studies included

**Fig. 3:**
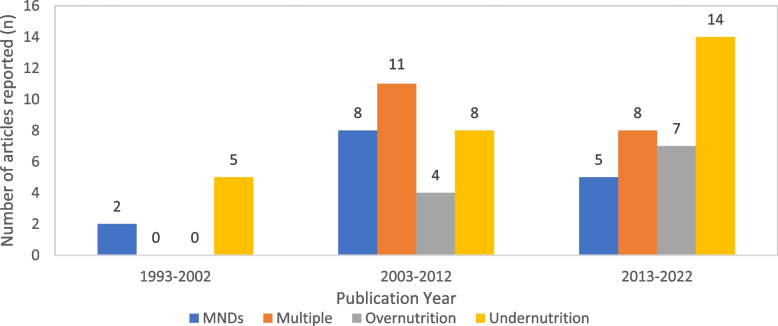
Number of studies reported by year of publication and types of malnutrition investigated

Of the 72 articles included in this review, there were 30 cross-sectional studies, 21 longitudinal studies, and 21 human intervention trials (including randomised/non-randomised, and controlled/non-controlled trials), with 27 focusing on undernutrition only, 12 on overnutrition only, 17 on MNDs only, and 16 investigating multiple forms of malnutrition. There were 37 studies involved children under 5, 24 studies for children aged 5–11 years old, and 11 studies for children aged 12–18 years old. The majority of the studies focused on children aged under 5 examined undernutrition as the outcome (*n* = 18), whereas for studies on children aged 12–18 years old, the majority studied overnutrition as the outcome (*n* = 7). Overall, undernutrition was more commonly studied (*n* = 27) compared to overnutrition (*n* = 11) (Table 2). Most of the studies were published after 2003, with 34 studies published in 2013–2022 and 31 studies published in 2003–2012, compared to 7 studies published in 1993–2002 (Fig. 3).

**Table 2:** Number of studies reported by age group and types of malnutrition investigated

	**Undernutrition**	**Overnutrition**	**MNDs**	**Undernutrition, overnutrition, and MNDs**	**Total**
< 5 years old	18	2	8	6	34
5–11 years old	9	2	6	8	25
12–18 years old	0	7	1	5	13
Total	27	11	15	19	72

If the age range overlapped with two age groups, the mean age of the study participants will be applied for categorisation

### Prevalence of different forms of malnutrition

The average age of participants was 5.3 years, and most of the participants were preschool or primary school-aged children (80%). Over 675,562 children were assessed over a period of 25 years, from 1988 to 2022. Table 3 shows the findings on the prevalence of malnutrition in Vietnamese children aged 2 months to 18 years old. Overall, the prevalence of overweight and obesity showed an increasing trend, especially in recent years. In Hanoi, the prevalence of overweight and obesity increased from 15.5% in 2013 to 21.2% in 2016 [19]. The prevalence of overweight and obesity in children aged 4–5 years old increased from 21.4% in 2002 to 36.8% in 2005 in Ho Chi Minh City [20]. A recent large-scale cross-sectional study in Ho Chi Minh City reported that overweight and obesity were more prevalent in primary school children, affecting 51.2% of them, compared to secondary school children (35.6%) and high school children (19.2%) [21]. Lower prevalence was observed in younger children aged 1–2 years old (10.7%) [22].

**Table 3:** Findings on the prevalence of malnutrition (undernutrition, overnutrition and micronutrient deficiencies) in Vietnamese children aged 0–18 years old

Authors and year	Study design & duration	Sample age	Sample size	Measurements	Key findings—prevalence	Risk of bias
** < 5 years old**						
Smuts et al. 2005 [2]	Baseline data of a double blinded, RCT	6–11 months	257	Weight, height, WAZ, HAZ, and WHZ Blood: serum levels of Hb, ferritin, retinol, and zinc	12.7% underweight 8.0% stunted 3.0% wasted 89.9% anaemic 20.2% retinol deficient 22.5% zinc deficient	Low
Khan et al. 2007 [23]	Repeat cross-sectional 1990–2005 (14 years)	0–5 years	357,396 1990 = 37,972 1994 = 37,654 2000 = 94,469 2002 = 91,921 2004 = 95,380	Weight, height, and BMI	Underweight decreased by 18.4% (from 45% to 26.6%) Stunting decreased by 25.8% (from 56.5% to 30.7%) Wasting decreased by 1.7% (from 9.4% to 7.7%)	Low
Nguyen et al. 2007 [24]	Cross-sectional	0–5 years	1,657	Blood: serum retinol and Hb Retinol deficiency = retinol < 0.70 μmol/L Anaemia: Hb < 110 g/L	12.0% retinol deficient 28.4% anaemic < 6 months old: 35.1% retinol deficient 61.7% anaemic	Low
Nhien et al. 2008 [25]	Cross sectional	1–6 years	243	Serum blood: copper, zinc, selenium, magnesium, retinol and Hb	86.9% zinc deficient 55.6% anaemic 11.3% retinol deficient 50.2% underweight 36.2% stunted 14.4% wasted	Low
Dieu et al. 2009 [20]	Repeated cross sectional study 2002–2005 (3 years)	4–5 years	1,162 2002 = 492 2005 = 670	Weight, height, BMI Underweight = WAZ < 5^th^ percentile Overweight/ obesity BMI > 23 kg/m^2^	36.8% obese (2005) 21.4% overweight 7.5% underweight Overweight/ obesity increased by 52.9% (p < 0.001)	Low
Vaktskjold et al. 2010 [26]	Prospective cohort study 2005–2006 (1 year)	0–1 year	237	Weight, HAZ, WLZ, and BMI-Z compared to WHO standards Blood: Hb (n = 189)	79% below median for weight-for-length 18.0% in 5^th^ percentile for length-for-age 9.6% in 5^th^ percentile for weight-for age 9.6% in 5^th^ percentile for weight-for-length 20.3% in 5^th^ percentile for BMI-for age 11.1% anaemic	Low
Laillou et al. 2013 [27]	Cross-sectional	0–5 years	532	Weight, height Blood: plasma calcium and vitamin D	21% vitamin D deficient 37% vitamin D insufficient 97% mild calcium deficient	Low
Nguyen et al. 2014 [28]	Cross-sectional	0–5 years	4,029	Weight and height	16.8% stunted 13.2% underweight 4.8% wasting	Medium
Lundeen et al. 2014 [29]	Longitudinal 8 years	1–8 years	1,830	Weight, height and HAZ	1 year: 21% stunted 5 years: 24% stunted 8 years: 19.2% stunted	Low
Giao et al. 2019 [22]	Prospective cross-sectional	1–2 years	768 (receiving vaccinations)	Weight, height, HAZ and BMI-Z	8.2% stunted 10.7% overweight/ obese	Medium
Kim et al. 2022 [30]	Cross-sectional	3–4 years	103	Weight, height, and BMI	22.3% overweight/ obese	Low
**5–11 years old**						
Hop et al. 1997 [31]	Longitudinal 1981–1994 (14 years)	0–10 years	212	Weight, height and feeding practices	Stunting at 21 months: 59.4% (male) & 58.3% (female)	Low
Hall et al. 2001 [32]	Cross-sectional 1998	7–11 years	588	Blood: Hb levels	13% anaemic	Medium
Mai et al. 2003 [33]	Cross-sectional 1999	7–9 years	348 Girls	Weight, height, LMAC, body fat and blood pressure	Rural: 21.8% underweight 13.5% stunted 11.5% wasted 0.0% obese Urban: 5.8% underweight 1.9% stunted 5.2% wasted 5.3% obese	Low
Tuan et al. 2008 [34]	Repeated cross sectional study 1992 – 2002 (10 years)	2–17 years	70,331	Weight, height, and BMI	Overweight/ obesity increased from 1.4% to 1.8% (p = 0.07) Underweight increased from 32.1% to 33.5% (p = 0.11)	Low
Nguyen et al. 2013 [8]	Cross-sectional SEANUTS	0.2–11.9 years	2,872	Weight, height, MUAC, waist and hip circumferences Blood: Hb, ferritin, vitamin A and D	14% stunted (< 5 years) 8.6% underweight (< 5 years) 4.4% thin (< 5 years) 15.6% stunted (5–11.9 years) 22.2% underweight (5–11.9 years) ~ 5% of overweight/obese children also stunted 38.65% anaemic (0.5–1.0 years) 18.25% anaemic (2–4.9 years) 12.47% anaemic (5–11 years) 7.75% retinol deficient (6–11.9 years)	Low
Huong et al. 2014 [35]	Cross-sectional	6 months-18 years	108	Weight, height and MUAC	19% wasted 13.9% stunted 0% obese 7% severe wasting at 6–59 months	Low
Le Nguyen et al. 2016 [36]	Cross-sectional 2011 SEANUTS	6–11 years	385	Blood: Hb, ferritin and red blood cell count	11.4% anaemic 5.6% iron deficient 0.4% ID anaemia	Low
Poh et al. 2016 [37]	Cross-sectional SEANUTS	2–15 years	2,016	Blood: serum vitamin D	11.1% vitamin D deficient 37.1% vitamin D insufficient 29.4% inadequate vitamin D	Low
Do et al. 2017 [19]	Longitudinal 2013–2016 (3 years)	3–9 years	2,602	Weight and height	Overweight increased from 9.1% to 16.7% Obesity decreased from 6.4% to 4.5%	Low
Thuc et al. 2019 [38]	Cross-sectional	6–11 years	155	Vitamin D deficiency: 25(OH)D < 50 nmol/L	23.9% vitamin D deficient	Medium
Le and Dinh 2022 [39]	Cross-sectional 2021	6–11 years	782	Height, weight and BMI-Z	14.32% obese 21.61% overweight	Low
**12–18 years old**						
Hong et al. 2007 [40]	Repeat cross- sectional study 2002 & 2004	11–16 years	3,687 2002 = 1,003 2004 = 2,684	Height, weight, and BMI-Z	Overweight significantly increased, 6.7% (p < 0.001) Obesity significantly increased, 1.4% (p < 0.001) Underweight decreased, 6.4% (insignificant) BMI significantly greater in 2004 than 2002 (p < 0.001)	Low
Van Nhien et al. 2009 [41]	Cross-sectional	11–17 years	245 girls	Weight, height and BMI Blood: Hb and trace element levels	20.7% undernourished 20.4% anaemic 26.5% zinc deficient	Low
Trang et al. 2012 [42]	Prospective cohort study 2004–2009 (5 years)	11–14 years	585	Weight and height	Overweight/ obesity increased by 7.6% (from 14.2% to 21.8%)	Low
Phan et al. 2020 [43]	Cross-sectional 2018	11–14 years	2,788	Weight and height using WHO and IOTF classification	17.4% overweight (WHO) 8.6% obese (WHO) 17.1% overweight (IOTF) 5.4% obese (IOTF)	Medium
Mai et al. 2020 [21]	Cross-sectional 2014–2015	6–18 years	10,949	Weight, height, BMI-Z and HAZ	Primary school children (6–13 years): 2.4% stunted 2.2% thin 24.3% overweight 26.9% obese Secondary school children (10–17 years): 3.8% stunted 4.6% thin 23.5% overweight 12.1% obese High school children (14–18 years): 7.9% stunted 6.0% thin 14% overweight 5.2% obese	Low

*BMI* body mass index, *BMI-Z* body mass index for-age-Z score, *HAZ* height-for-age-Z score, *Hb* haemoglobin, *IOTF* international obesity task force, *LAZ* length-for-age-Z score, *LMAC* left mid arm circumference, *MUAC* mid upper arm circumference, *ND* no data, *WAZ* Weight-for-age-Z score, *WHO* World Health Organization, *WHZ* weight-for-height-Z score, *WLZ* weight-for-length-Z score, *25(OH)D* 25-hydroxy vitamin D

The prevalence of undernutrition has gradually decreased since the 1990s. A large-scale longitudinal study reported that from 1990 to 2005, the prevalence of stunting in children aged under 5 years old has reduced from 56.5% to 30.7%, wasting from 9.4% to 7.7%, and underweight from 40.0% to 26.6% [23]. In 2014–2015, it is reported that 2.4%, 3.8%, and 7.9% of the primary, secondary, and high school aged children were stunted, and 2.2%, 4.6%, and 6.0% were wasted, respectively [21, 22]. Other studies also reported that the prevalence of stunting was higher in children aged under 6 years old (16–36%) [21, 22], compared to children aged 6 years old and above (14%) [44]. There were more underweight children in younger age groups, affecting 50% of children aged 1–6 years old [25] compared to 22% in children aged 7–9 years old [44].

The most commonly reported MNDs were iron, zinc, and vitamin A. The prevalence of each MND differed between studies, depending on the micronutrient investigated, age and location. MNDs were also more frequently investigated in younger children aged < 6 years old compared to children aged over 6 years old. Anaemia was more prevalent in younger children aged below 6 years old (28.4–38.6%) [8, 24, 35, 45] compared to older children aged 6–11 years old (11.4%) [24, 35]. Prevalence of iron deficiency varied greatly from 11% in the nationally representative South East Asia Nutrition Surveys (SEANUTs) to 90% in certain rural districts [36]. Retinol deficiency was of less concern compared to other MNDs, ranging from 8% in 0.2–11.9 years old [2, 8] to 20% in 6–11 months [2, 8]. Zinc deficiency varied largely from 23% in infants aged 6–11 months, to 87% in children aged 12–72 months [2, 25]. In 2009, a cross sectional study reported that almost 58% of children aged under 5 years old were affected by vitamin D deficiency [27]. Similar finding was found SEANUTs I which reported that vitamin D deficiency was found in 48% of the children aged 0–11 years old [37], whereas a smaller-scale study reported that vitamin D deficiency was affecting 29% of children of same age group [38]. Only one study reported the prevalence of calcium deficiency, with 97% of 0–5 years old being mildly calcium deficient [27].

### Determinants of different forms of malnutrition

Out of a total of 31 records, 36% were nationally representative, 36% were in urban areas, 12% were in rural areas, 9% were in urban and rural areas (but not nationally representative), and 6% were in mountainous areas (Table 4). This included 575,193 children, with ages ranging from 0–18 years old. The majority of these studies were of preschool and primary school-aged children (51%), followed by middle and high school-aged children (24%). This includes longitudinal (*n* = 9) and cross-sectional design (*n* = 18). Most studies collected anthropometric measurements (weight, height, and BMI) and questionnaires asking about lifestyle factors such as physical activity status or screen time. Demographic information was also collected in most questionnaires, including economic status, parents’ education level, occupation, or family size. The studies examined the determinants of undernutrition (41%, *n* = 14) [11, 23, 28, 35, 44–53], overnutrition (24%, *n* = 10) [30, 39, 40, 54–60], undernutrition and overnutrition (18%, n = 6) [21, 33, 61–64], MNDs (9%, *n* = 3) [32, 65, 66] all three types of malnutrition (6%, *n* = 2) [8, 67], and undernutrition and MNDs (3%, *n* = 1) [41].

**Table 4:** Findings on the determinants associated with different forms of malnutrition in Vietnamese children aged 0–18 years old

Authors and year	Study duration & design	Sample age	Sample size	Measurements	Key findings – risk factors / determinants	Risk of bias
Hanieh et al. 2015 [44]	Prospective longitudinal	6 months	1,046	Length, weight, and LAZ	Positive association between infant LAZ scores at 6 months and maternal BMI (coefficient 0.04 kg/m^2^, CI = 0.01–0.07), weight gain during pregnancy (0.04/kg, CI = 0.01–0.06) and maternal ferritin concentration (− 41.5 g/twofold increase in ferritin, CI = − 78 to − 5.0) Inverse association between maternal 25-(OH)D concentration and infant LAZ scores (coefficient − 0.06 per 20 nmol/L, CI = -0.11 to -0.01) No association between maternal iodine status & infant LAZ	Low
Vaktskjold et al. 2010 [26]	Prospective cohort 2005–2006 (1 year)	0–1 year	237	Weight, height, LAZ, WHZ, and BMI-Z	Lower LAZ (β = -2.2, CI = -4.0 to -0.5) and WAZ (-0.5, CI = -1.0 to -0.1) were statistically associated with living rurally	Low
Hien and Hoa 2009 [11]	Cross-sectional	0–3 years	383	Weight and height	Positive association between being underweight and rural living region (OR = 2.22), minority ethnicity (OR = 1.74), mother’s occupation-housewife (OR = 7.91), household size ≤ 4 (OR = 3.07), underweight mother (OR = 1.95), number of children in the family ≥ 3 (OR = 3.35), low birth weight < 2500 g (OR = 7.99), exclusive breastfeeding duration < 6 months (OR = 4.41) and initiation of breastfeeding after 1 hour (OR = 2.54)	Medium
Tran 2008 [45]	Cross-sectional	0–3 years	547	Height, weight and questionnaire	Positive association between fathers not taking children to a medical facility for immunisation and being underweight or stunted (OR = 1.75, CI = 1.07–2.87)	Low
Khan et al. 2007 [23]	Repeat cross-sectional 1990–2005 (14 years)	0–5 years	357,396	Weight, height and BMI	Higher prevalence of underweight, stunting and wasting in rural and mountainous areas than urban areas. Larger rates of reduction of underweight, stunting and wasting in urban areas, than rural and mountainous areas Household size and being a male are all both positively associated with being stunted (β = -0.1543, p = 0.0001)	Low
Nguyen et al. 2014 [28]	Cross-sectional	0–5 years	4,029	Weight and height Maternal CMD	Maternal CMD was positively associated with underweight children (OR = 1.27, CI = 1.01–1.61). Low birth weight was positively associated with stunting (OR = 3.71, p < 0.001), underweight (OR = 3.96, p < 0.001), and wasting (OR = 3.61, p < 0.001). Poor household wealth was positively associated with underweight (OR = 1.99, p < 0.01)	Low
Huong et al. 2014 [35]	Repeat cross–-sectional—seasons	2–4.9 years	853	Weight, height and BMI	Summertime is positively associated with being underweight (p < 0.05) and stunted (p < 0.05)	Low
Chen 2021 [51]	Young Lives Study Longitudinal 2002–2006 (4 years)	1–6 years	2,000	Weight, height, WAZ, HAZ and WHZ	Negative association between malnutrition and family size; having one additional child is associated with declines of the first child’s HAZ (0.49 SD) and WAZ (0.57 SD)	Medium
Kim et al. 2022 [30]	Cross-sectional	3–4 years	103	Weight, height, physical activity and sedentary behaviour	Children not meeting screen time guidelines were at a higher risk of being overweight/ obese but was not significant (OR = 0.94, p = 0.904)	Low
Lavin et al. 2017 [49]	Longitudinal cohort study 8 years	0–8.5 years	1,812	Weight, height and HAZ	Positive associations with moderate/severe stunting were found in low birth weight (OR = 0.114, p = 0.001), food shortages (OR = 0.048, p < 0.001), rural location (OR = 0.068, p < 0.001), decreasing wealth (OR = 0.080, p = 0.008) and ethnic minority (OR = 0.077, p < 0.001)	Low
Bennett et al. 2015 [46]	Young Lives Longitudinal Cohort study 2001–06	1–8 years	1,961	Weight, height, questionnaire and risk of maternal CMD	Maternal CMD is positively associated with stunting at age 1 (ARR = 1.24, CI = 1.03–1.62), age 8 (1.22, CI = 1.03–1.45)	Low
Dearden et al. 2017 [48]	Longitudinal	1–8 years	1,905	Height, weight and BMI-Z	Improved access to water at 1 year old was negatively associated with stunting at 1 (RR = 0.27–1.20), 5 (RR = 0.25–1.17) and 8 (RR = 0.25–1.42) years old	Low
Huynh et al. 2011 [63]	Cohort study 2005–2006	4–5 years	526	Weight, height, SSF: SSFT, TSFT and suprailia Questionnaire: physical activity level	BMI and SSF were negatively associated with neighbourhood safety for boys (β = -0.80, CI = –1.53 to –0.08) and girls (β = -0.59, CI = -1.16 to -0.01) Boys increasing BMI was positively associated with both parents being overweight (β = 1.18, CI = 0.21–2.16) Availability of food at home was associated with increased BMI in girls (β = 1.23, CI = 1.91–0.55) but not boys	Medium
Nguyen et al. 2013 [8]	SEANUTS multi-stage cluster-randomised sampling	0.2–11.9 years	2,872	Weight, height, mid-upper arm circumference, waist and hip circumferences Blood: Hb, serum ferritin, vitamin A and D	Positive association between undernutrition and rural habitation (p < 0.05) Positive association between overnutrition and urban habitation (p < 0.05) Ferritin: significantly higher levels in urban girls than urban boys (p < 0.05) and significantly higher levels in urban children than rural children (p < 0.05) aged 6–11.9 years	Low
Nguyen et al. 2021 [52]	Cross-sectional, follow up	6–7 years	1,579	Weight, height and BMI Maternal weight, height and BMI	Preconception maternal nutritional status is positively associated with child attained size at age 6–7. Child HAZ was positively associated with maternal height (+ 0.28 SD) and BMI (+ 0.13 SD), and faster linear growth at age 6–25 months (β = 0.39–0.42)	Low
Trinh et al. 2021 [53]	Longitudinal	5 months – 13 years	2,000	Rainfall data (flooding, drought), weight, height, BMI, HAZ and WAZ	Positive association between flooding and being stunted (RR = 0.122, p < 0.01) and underweight (RR = 0.067 p < 0.01). Positive association between droughts and stunting (RR = 0.127, p < 0.01) Rainfall shocks can impact parental mental health, increasing the probability of child being underweight by 0.976 (p < 0.001)	Medium
Hoang et al. 2019 [67]	Cross-sectional	6–9 years	839	Weight and height Blood: Hb and mean corpuscular volume	Underweight, stunting and wasting were all positively associated with anaemia (p < 0.004), specifically normocytic anaemia (p < 0.006) No significant association between anaemia and demographic indicators or socio-economic indicators	Low
Nguyen 2022 [50]	Cross-sectional	0–15 years	158,019	Height, weight, HAZ and WAZ	Preschool attendance is negatively associated with prevalence of underweight (p = 0.079) and stunting (p = 0.079) at 2–15 years	Medium
Krishna et al. 2015 [47]	Longitudinal–	6 months—15 years	2,489	Weight, height and HAZ	Wealth index is positively associated with growth in children	Low
Mai et al. 2003 [33]	Cross-sectional 1999	7–9 years	348 girls	Weight, height, WAZ, HAZ, WHZ and LMAC	Positive association between rural living and being underweight (OR = 4.5, p < 0.001), stunted (OR = 7.9, P < 0.001), wasted (OR = 2.4, p = 0.039) or undernourished (OR = 3.0, p = 0.045)	Low
Van Lierop et al. 2008 [62]	Cross-sectional	6–10 years	2,631	Weight, height and waist circumference	21.4% were stunted, and higher prevalence was found in rural regions (23.8% vs 17.3%, p < 0.001) Living in urban areas is positively associated with being overweight (4.6% vs 1.6%, p < 0.001)	Low
Le and Dinh 2022 [39]	Cross-sectional, two-stage cluster random sampling	6–11 years	782	Questionnaire, weight, height, and BMI-Z	Significant positive association between male and childhood obesity (OR = 2.48, p < 0.0001) Positive association between overweight/ obesity and children who live with only their father (OR = 11.96, p = 0.0219), transport to school being inactive (motorbike/car/bus) (OR = 1.58, p = 0.0096) and mother’s occupation being white collar (OR = 1.56, p = 0.004)	Low
Hung et al. 2005 [65]	Longitudinal 1997–2000 (3 years)	0–17 years	2,767	Blood: Hb and ferritin Malaria and intestinal helminth infection (worms)	Malaria is significantly positively associated with anaemia (OR = 2.408, p = 0.0006) No significant association between intestinal helminth & anaemia	Medium
Hall et al. 2001 [32]	Cross-sectional	7–11 years	588	Blood: Hb levels	Anaemia is positively associated with boys: Aged 7–11 years (RR = 1:07, CI = 1.10–1.13) Aged 12–14 years (1.18, CI = 1.12–1.24) Aged ≥ 15 years (1.30, CI = 1.16–1.46)	Medium
Mai et al. 2020 [21]	Cross-sectional 2014–2015	6–18 years	10,949	Weight and height	Positive association between overweight status, urban living (p < 0.001), and male (p < 0.001) Positive association between underweight status and rural living (p < 0.001)	Low
Trang et al. 2012 [56]	Longitudinal study 2004–2009 (5 years)	11–14 years	759	Weight, height, BMI, questionnaire: level of physical activity and socio-economic status	Levels of ‘moderate-to-vigorous physical activity’ are negatively associated with overweight/ obesity (RR = 0.60, CI = 0.53–0.67)	Low
Tang and Dibley 2022 [59]	Longitudinal	10–15 years	482	Weight, height and BMI SSF: SSFT and TSFT	Male at higher risk for higher BMI than girls (p = 0.006) Inactive adolescents at higher risk of gaining weight than active adolescents; TSFT (RR = 1.43, CI = 1.22–1.67), SSFT (1.09, CI = 1.00–1.18) and BMI (1.06, CI = 1.02–1.10)	Low
Tang et al. 2020 [57]	Cross-sectional	10–15 years	2,660	Weight, height and BMI SSF: SSFT and TSFT	Overweight status was positively associated with boys (p < 0.0001) TSFT & SSSF significantly higher in girls than boys (p < 0.0001)	Low
Nguyen et al. 2022 [58]	Cross-sectional	11–15 years	2,660	Weight, height and consumption of sugar-sweetened beverages (SSBs)	Negative association between overweight/ obese status and consumption of milk based SSBs. Every kcal more of fresh milk with sugar & condensed milk, can reduce the obesity odds of 0.005 (CI = 0.002–0.008)	Low
Hong et al. 2007 [40]	Repeated cross-sectional 2002–2004	11–16 years	3,687	Weight, height and BMI	Positive association between increase in overweight/ obesity and male: 113% increase (p < 0.001) Significant difference in the increase in prevalence by gender: obesity and overweight in males increased by 113%, with only a 39% increase in girls Poorer households showed smaller increase in obesity/overweight at 33%, compared to wealthier households at 77–124%	Low
Tang et al. 2007 [61]	Cross-sectional	11–16 years	1,504	Weight, height and BMI-Z	Being male is positively associated with being underweight (p = 0.001) Non-significant association between being male and being overweight or obese (p = 0.074) Positive association between living in wealthy urban districts and being overweight/ obese (p < 0.001)	Low
Trang et al. 2009 [55]	Cross-sectional	11–16 years	2,684	Weight, height, questionnaire: physical activity levels, and family characteristics	Being overweight is positively associated with physical inactivity (OR = 2.5, CI = 1.9–3.2), passive transport to school (OR = 4.2, CI = 3.3–5.2), no recess exercise (OR = 1.3, CI = 1.1–5.6), time spent playing video games (OR = 2.3, CI = 1.7–3.1), or watching television (OR = 1.5, CI = 1.2–1.9)	Low
Van Nhien et al. 2009 [41]	Cross-sectional	11–17 years	245 girls	Weight and height Blood: serum Hb and selenium	Anaemia is positively associated with selenium deficiency (OR = 5.36, CI = 2.57–11.18), being underweight (2.72, CI = 1.37–5.37) and years of age (1.35, CI = 1.14–1.59) in girls	Low
Tran et al. 2017 [64]	Cross-sectional	12–17 years	1,851	Weight, height and memory tests	No significant association between child maltreatment and overweight or underweight status Underweight status is negatively correlated with working memory (r = -0.07, p < 0.01) and academic performance (r = -0.08, p < 0.01) Overweight status is positively associated with male (OR = 1.39, p = 0.00) and negatively associated with rurality OR = 0.66, p = 0.04)	Low

*BMI* body mass index, *BMI-Z* body mass index for-age-Z score, *CI* confidence interval, *CMD* common mental disorder, *HAZ* height-for-age-Z score, *Hb* haemoglobin, *MUAC* mid upper arm circumference, *LAZ* length-for-age-Z score, *LMAC* left mid arm circumference, *OR* odds ratio, *RR* relative risk, *SSF* skinfold thickness, *SSSF* subscapular skinfold thickness, *TSFT* tricep skinfold thickness, *WAZ* weight-for-age-Z score, *WHZ* weight-for-height-Z score

Generally, more studies reported the determinants for undernutrition in younger children and overnutrition in older children. Child undernutrition was highly associated with poverty or low socioeconomic status related indicators such as those living in rural (OR = 2.22–4.50) [11, 33] or mountainous areas [23], food insecurities [49], low household wealth [28, 49], ethnic minorities [11], preschool attendance [50], poor immunisation coverage [45], and disasters such as flooding and drought [53] (*distal factors*); as well as increased household size [23], and poor hygiene and drinking water sources [48] (*intermediate factors*). Early life nutrition including low birth weight [11, 28, 49], poor maternal nutrition (e.g., undernourished mother) [11, 44, 52], and poor breastfeeding and feeding practices [11] (*proximal factors*), were also found to be associated with increased risk of undernutrition.

Males were more likely to be stunted/underweight [23], overweight/obese [39, 40, 64], and anaemic [32] than females (*proximal factors*) (Table 5). Moreover, the risk of being overweight or obese was 1.39 times higher in physically inactive children [64], and 2.5 times higher in those living in urban areas (*p* < 0.05) [32]. Maternal nutrition was also associated with both child undernutrition and overnutrition (*intermediate factors*). Other determinants of being overweight include living in a wealthy household [40], inactive mode of transport to school [8], having overweight parents [63], and low food availability at home in girls only [63]. Few studies reported the determinants of MNDs, nonetheless iron deficiency was found to be associated with malaria in ethnic minorities [65]; selenium deficiency and being underweight in adolescent girls [41]; and being stunted and underweight in primary school children from the rural areas [67] (*proximal factors*).

**Table 5:** Number of studies reporting shared determinants of different forms of malnutrition in Vietnamese children aged 0–18 years old

Malnutrition Type	Rurality	Socioeconomic status: poorer	Food availability	Maternal nutrition	Gender (Male)	Physical Activity	Low birth weight	Ethnic minority	Household size	Maternal CMD
Undernutrition	+ + + + + + + +	+ + +	–	–	+ +		+ + +	+ +	+ + +	+ +
Overnutrition	- - - - -	-	+/-	+	+ + + + + +	- - - -				
Iron deficiency					+					

Number of + and – refers to the number of studies reporting relevant associations. + Positive association,—Negative association, ± conflicting results. *CMD *common mental disorder

### Interventions and its impacts on addressing different forms of malnutrition

Twenty one articles reported intervention studies addressing different forms of malnutrition, mainly focusing on undernutrition and MNDs (Table 6). No intervention study was found addressing overnutrition. These include 4 nutrition sensitive and 17 nutrition specific programmes. Approximately 8,087 children participated in the intervention studies from 1996 to 2015, in addition to a few large-scale studies evaluating the impact of the national government programme on children’s nutritional status. The duration of these studies ranged from 30 days to 2 years, and all studies were randomised control trials (RCT) except for one non-random pragmatic trial, which was carried out without a control/ placebo treatment [68]. The interventions mainly targeted younger aged children ranged from 0–8 years old.

**Table 6:** Findings on the interventions and its impacts on addressing different forms of malnutrition in Vietnamese children aged 0–18 years old

Authors and year	Study design & duration	Sample age	Sample size	Inclusion criteria	Intervention group *Control group*	Measurements	Key findings – results and effectiveness	Risk of bias
**Nutrition sensitive intervention studies**								
Hop and Khan 2002 [3]	Follow-up of national nutrition strategy 1995–2000	0–5 years	National	Child living in included area	National Plan of Action for Nutrition (NPAN), poverty reduction, infrastructure improvement, financial support, agriculture and aquaculture extension, health care, credit & education	Weight and height	Stunting decreased from 58% to 37.3% Underweight decreased from 51.5% to 25%	Medium
Mackintosh et al. 2002 [69]	Follow-up study to assess effectiveness of PANP (1993–1995) after 3 4 years (1998–1999)	4–6 years (older) 1–3 years (younger group)	55	Families who previously participated in the PANP study and 1 younger child who had not received any PANP exposure Control group: no previous exposure to PANP	Poverty alleviation and nutrition program (PANP): growth monitoring and promotion, positive deviance inquiry, nutrition education and rehabilitation programme, and revolving loan program (n = 46 household, 142 children) *Control group: no intervention* *(n* = *25 household)*	Weight, height and WAZ	After 24 months: severe malnutrition (using WAZ) had reduced from 23 to 6% No significant difference in WAZ between the groups Intervention group were ‘nutritionally better off’, had better feeding habits and weaning practices	Medium
Watanabe et al. 2005 [70]	Uncontrolled trial and follow up (2004) 5 years total	Intervention: 4–5 years Follow up: 6.5–8.5 years	313	Living in a commune with a high prevalence of malnutrition, poor socioeconomic conditions, no prior intervention programme, and leaders being interested in the project	Nutrition intervention group: including growth monitoring, nutrition education rehabilitation programme, nutrition-seeking and health-seeking behaviours, feeding children locally available nutritious foods, antenatal care, home gardening, savings & credit programme (n = 172) Nutrition programme (as above) & early childhood development (ECD) group: follow up to the prior intervention Parental training: care and development. (n = 141)	Height, weight, HAZ, WAZ, WHZ, maternal and household characteristics	No statistically significant differences between intervention groups for anthropometric measures, or levels of stunting, wasting or underweight status Longitudinal results: significant decrease in stunting prevalence in both the nutrition intervention group (13.4%, p < 0.01) and ECD & nutrition group (16.3%, p < 0.01). Severe stunting was only reduced in the ECD & nutrition group by 7.8% (p < 0.01)	Medium
Pachon et al. 2002 [71]	Longitudinal, RCT 2 years	5–25 months	239	Malnourished children matched with healthy children	Save the Children: positive deviance children interviewed to find key ‘good foods’ & behaviours. This included bimonthly nutrition rehabilitation for 9 months to identify ‘good foods,’ increase food quantity, and promote breastfeeding; and monthly growth monitoring and promotion sessions for 2 years (n = 119) *Control group: no intervention (n* = *119)*	Weight, height, BMI, WAZ, HAZ, WHZ and breastfeeding status	At 12 months, intervention children consumed 20% more food than control group (p < 0.01), and were fed more times a day than the control group (p < 0.01) No statistically significant results for WAZ at 12 months At months 2–6, for children < 15 months, 44.6% control group were undernourished compared to 68.8% (p < 0.05) However, children > 15 months, the intervention group (45.2%) had more well-nourished children than the control group (29.6%, p < 0.01)	Low
**Nutrition specific intervention studies**								
Wieringa et al. 2007 [72]	Double blinded, RCT 6 months	4–6 months	784	No chronic or severe illness, severe clinical malnutrition, anaemia, congenital anomalies	Supplementations for 7 days/week: Zinc (Zn): 10 mg/day (n = 196) Iron (Fe): 10 mg/day (n = 196) Iron + zinc: 10 mg each/day (n = 196) *Control group: unfortified syrup (n* = *196)*	Weight, height, BMI, WAZ, HAZ, WHZ Hb, SF and serum zinc	The Fe and Fe + Zn groups had significantly higher levels of Hb and SF, and lower prevalence of anaemia, than the Zn and placebo groups (p < 0.0001). Iron supplementation significantly increases Hb levels (p < 0.0001) The Zn and Fe + Zn groups had significantly higher levels of zinc than the placebo and Fe groups (p < 0.0001). After baseline value adjustment, Zn levels were significantly higher in the Zn group compared to the Fe + Zn group (p = 0.02) Zinc supplementation had a negative effect on Hb concentrations, independent of iron supplementation (-2.5 g/L, p < 0.001, p-interaction = 0.25)	Low
Hall et al. 2007 [73]	Cluster randomised trial (CRT), 17 months	6 years	1,080	Children in primary schools who had taken part in a school feeding programme (fortified biscuits & milk)	Intervention group: fortified biscuits and milk, total 300 kcal. Once a day, 5 times a week. Deworming. Nutrition and hygiene information (n = 360) *Control group: no intervention* *(n* = *720)*	Weight, height, BMI, WHZ, WAZ and HAZ	The intervention group gained significantly more weight (3.19 kg vs 2.95 kg, p < 0.001) and height (8.15 cm vs 7.88 cm, p = 0.008) than the control group. After controlling for other limiting factors, the intervention programme was statistically significant for weight gain (p = 0.024), and the most undernourished children at baseline gained the least weight	Low
Hanieh et al. 2014 [74]	Cluster randomised trial (CRT) & follow up, 1 year	6 months	1,175	Pregnant women	IFA: Iron + folic acid supplement daily (60 mg iron + 0.4 mg folic acid) (n = 395) IFA: Iron + folic acid supplement twice weekly (60 mg iron + 1.5 mg folic acid) (n = 399) MMN: Multiple micronutrient supplement + Iron and folic acid, twice weekly (60 mg iron + 1.5 mg folic acid + 13 other micronutrients) (n = 381)	Birthweight, length, and weight	No difference in birth weight as well as infant LAZ at 6 months of age in the twice weekly IFA group compared to the daily IFA group (MD 20.14, CI = 20.29–0.02), nor in the twice weekly MMN group compared to the daily IFA group (MD 20.04, CI = 20.20–0.11)	Low
Hanieh et al. 2013 [75]	CRT and follow up 1 year	6 months	Follow up: 891	Pregnant women	IFA: Iron + folic acid supplement daily (n = 395) (60 mg iron + 0.4 mg folic acid) (n = 395) IFA: Iron + folic acid supplement twice weekly (n = 399) (60 mg iron + 1.5 mg folic acid) (n = 399) MMN: Multiple micronutrient supplement + Iron and folic acid, twice weekly (n = 381) (60 mg iron + 1.5 mg folic acid + 13 other micronutrients) (n = 381)	LAZ, head circumference, and HAZ	Follow up: Inverse association between maternal 25-OHD status and infant HAZ at 6 months (OR = -0.09, CI = -0.12 to -0.02)	Low
Hop and Berger 2005 [76]	Double blinded, RCT 6 months	6–12 months	306	Not severely wasted, not born prematurely	DDM: daily multiple micronutrient supplement (15 micronutrients including iron) (n = 76) WMM: weekly multiple micronutrient supplement (15 micronutrients including iron) (n = 77) DI: daily iron supplement (Daily adequate intake) (n = 75) *Control group (P): daily placebo* *(n* = *73)*	Weight, length, LAZ, WAZ, plasma Hb, ferritin, zinc, riboflavin, retinol, tocopherol, and homocysteine	LAZ and WAZ worsened significantly in all groups, apart from LAZ in the DDM group which was significantly less than in the P and WMM groups (p = 0.001) Hb levels increased significantly more in the DMM group (mean = 16.4 g/L, CI = 12.4–20.4) than the P group (mean = 8.6 g/L, CI = 5.0–12.2) PF levels increased significantly more in the DMM and DI groups than the P and WMM groups	Low
Huy et al. 2009 [77]	Non-random, non-controlled pragmatic trial 2 years	0–2 years	586	Pregnant women	1: Iron (60 mg) + folic acid supplement (400 µg) (n = 211) 2: Multiple-micronutrient supplement (n = 203) 3: Gender training – maternal care from the family and community during pregnancy, and multiple-micronutrient supplement (n = 172) All: nutrition education- encouraging more frequent eating during pregnancy	Baby birth weight (LBW < 2500 g) At 2 years: weight and height	Average birth weight was higher in the two groups receiving multiple-micronutrient supplements than the group receiving iron = folic acid (2: + 166 g 3: + 105 g) than those receiving iron + folic acid (p < 0.05) LBW prevalence was lower in groups 2 & 3 than in group 1 (4.0%, 5.8% and 10.6% respectively, p < 0.05) At 2 years: children were taller in groups 2 & 3 than group 1 (p < 0.05) and stunting rates were ~ 10% lower (p < 0.05). No statistical significance for weight indicators	Medium
Le et al. 2007 [78]	RCT 6 months	6–8 years	425	Anaemic children	Iron fortified noodles, 10.7 mg/day (n = 86) Iron fortified noodles (10.7 mg/day) + mebendazole (n = 79) Mebendazole (deworming drug) (n = 79) Iron tablet (dose not reported) + mebendazole (n = 83) *Control group: Placebo* *(n* = *82)*	Iron status: Hb, SF, sTfR, and haemoglobinopathies analysis Inflammation: C-reactive protein Parasite infection status and immunoglobulin E (IgE)	Hb concentration improved, and anaemia prevalence reduced in all groups (p < 0.001). Iron fortification significantly increased levels of Hb, SF and body iron (p = 0.037, p < 0.001 and p < 0.01, respectively), compared to just deworming and the placebo. Deworming showed no increased effect on Hb, iron status or IgE level compared to iron fortification	Low
Le et al. 2006 [79]	RCT 6 months	6–8 years	425	Anaemic children	Iron fortified noodles, 10.7 mg/day (n = 86) Iron fortified noodles (10.7 mg/day) + mebendazole (n = 79) Mebendazole (deworming drug) (n = 79) Iron tablet (dose not reported) + mebendazole (n = 83) *Control group: Placebo* *(n* = *82)*	Hb, SF, sTfR, and haemoglobinopathies analysis, CRP, parasite infection status, and immunoglobulin E (IgE)	Iron supplementation was more efficient than fortification to treat anaemia for all iron markers: Supplementation (Hb 6.19 g/L, p = 0.001; SF 117.3 μg/L, p = 0.001; and body iron 4.37 mg/kg, p = 0.001) compared to fortification (Hb 2.59 g/L, p = 0.07; SF 23.5 μg/L, p = 0.006; and body iron 1.37 mg/kg, p = 0.001)	Low
Ninh et al. 1996 [80]	Double blinded, RCT 5 months	4–36 months	146	Growth-retarded children, paired to healthy children	Zinc supplementation (10 mg) daily (n = 73) *Control group: Placebo (n* = *73)*	Weight, height, WAZ, HAZ plasma circulating insulin-like growth factor (IGF-I)	Zinc supplementation increased weight by 0.5 kg (± 0.1 kg, p < 0.001) and height by 1.5 cm (± 0.2 cm, p < 0.001)	Low
Pham et al. 2020 [81]	RCT 6-month intervention 18-month follow-up	5 months	426	Singleton, breastfed infants Severe anaemia (Hb < 70 g/L)	FF: instant fortified flour, daily for 6 months containing 11 vitamins & 12 minerals (n = 157) FC: complementary fortified food, daily for 6 months containing 11 vitamins & 12 minerals (n = 135) *Control group (C group): no intervention (n* = *134)*	Micronutrient status: Hb, PF sTfR, zinc, and retinol	Iron deficiency and iron deficiency anaemia were lower in the FF (13.4% and 6.7%) and FC (15.2% and 3.8%) groups compared to the C group (57.5 and 37.5%, p < 0.0001)	Low
Phu et al. 2012 [82]	RCT 6-month intervention 18-month follow-up	5 months	377	Severe anaemia (Hb < 70 g/L)	FF: instant fortified flour, daily for 6 months containing 11 vitamins & 12 minerals (n = 135) FC: complementary fortified food, daily for 6 months containing 11 vitamins & 12 minerals (n = 114) *Control group: no intervention (n* = *128)*	Micronutrient status: Hb, PF, sTfR, zinc, and retinol	Retinol & zinc concentrations didn’t differ significantly among groups. Zinc deficiency was significantly lower in the FF group (36.1%) than C group (52.9%, p = 0.04)	Low
Thach et al. 2015 [83]	Cluster randomised trial (CRT) 9-month intervention 25-month follow-up	6 months	426	Pregnant women	Daily iron-folic acid (IFA) (60 mg elemental iron and 0.4 mg folic acid) (n = 34 communes) Twice weekly IFA (60 mg elemental iron and 1.5 mg folic acid) (n = 35 communes) Twice weekly multiple-micronutrient, iron and folic acid (60 mg elemental iron, 1.5 mg folic acid and MMN) (n = 35 communes)	Weight, length, LAZ and WAZ	The OR of anaemia was significantly lower among infants in the daily IFA (OR = 0.31, CI = 0.22–0.43), weekly IFA (0.38, CI = 0.26–0.54) and MMN (0.33, CI = 0.23–0.48) compared to groups in the observational study	Low
Berger et al. 2006 [84]	Double blinded, RCT 6 months	4–7 months	915	Breastfed infants aged 4–7 months who free from chronic/ acute illness, severe malnutrition, or congenital abnormalities	Fe-group: daily dose of 10 mg of iron as ferrous sulfate (n = 201) Zn-group: daily dose of 10 mg zinc as zinc sulfate (n = 195) Fe–Zn group: a daily dose of 10 mg iron þ 10 mg zinc (n = 190) *Control group: Placebo: a dose of 100 000 IU of vitamin A was given to all infants to avoid VAD* *(n* = *198)*	Stunting HAZ < -2 z-scores; wasting WHZ < -2 z-scores; underweight HAZ < -2 z-scores; anaemia = Hb < 110 g/l; low Fe stores = SF < 12 mg/l; low Zinc = Zinc < 9.9 mmol/l; IDA = simultaneous low SF and anaemia	Hb and SF levels significantly increased in both Fe and Fe + Zn groups (22.6 and 20.6 g/l for Hb; 36.0 and 24.8 mg/l for SF, respectively) compared to Zn and placebo groups (Hb: 6.4 and 9.8 g/l; SF: 18.2 and 16.9 mg/l, p < 0.0001). Zn increased more in Zn group (10.3 mmol/l) than in Fe + Zn group (8.0 mmol/l, p < 0.03), and Fe and placebo groups (1.6 and 1.2 mmol/l, p < 0.0001). Adding iron to zinc supplements negates the positive effect that sole zinc supplementation had on weight gain (WAZ) (p = 0.0004) and serum zinc (p = 0.02) showing a significant interaction between zinc and iron co-supplementation	Low
Vuong et al. 2002 [85]	Controlled trial 30 days	31–70 months	185	Children with low Hb concentration (100–120 g/L)	Vitamin A supplementation: using Momordica coincidences (gac) fruit (locally available) 1: fruit & rice = 3.5 mg β-carotene (n = 62) 2: powder & rice = 5.0 mg synthetic β-carotene (n = 60) 3: Plain rice, no fortification (n = 63)	Weight, height, HAZ, WAZ and plasma β-carotene and Hb concentration	β-carotene concentrations significantly higher in groups 1 & 2 than group 3 (p < 0.0001) Plasma retinol concentration significantly higher in group 1 (p = 0.0053) than group 2 and (p = 0.0053) group 3 Plasma retinol concentrations were significantly higher in group 1 than group 2 (p = 0.0053) and group 3 (p = 0.006) Hb concentrations increased in all 3 groups. In anaemic children, Hb levels were significantly lower in group 3 than group 1 (p = 0.017), but not than group 2	Medium
Xuan et al. 2013 [86]	RCT 5 months	18–36 months	334	Not breastfed, no congenital or chronic diseases, and not consuming prebiotics or probiotics	Intervention group: GAU 1 + milk-isocaloric and isoprotic gum, containing synbiotics, and fortified with vitamins A, C and E, and minerals zinc and selenium, and docosahexaenoic acid (n = 150) *Control group: Fortified gum of sufficient protein, carbohydrates, fats, vitamins, and minerals (n* = *184)*	Height and weight gain, anaemia, zinc, and vitamin A deficiencies	The growth parameters of the intervention group increased significantly more than the control group: Weight (+ 0.43, p < 0.01) Height (+ 1 cm, p < 0.01) and BMI Z-score (+ 0.015, p < 0.05) MNDs were reduced in both the intervention and control groups, more in the intervention groups, but non-statistically significant, anaemia by 14.9% (p = 0.63), vitamin A by 9.5% (p = 0.05) and zinc by 13.6% (p = 0.44)	Low
Nguyen et al. 2021 [87]	Uncontrolled trial, 6 months	6–14 years	151	Children from 5 schools in Can Tho with Vitamin D deficiency/ insufficiency/ low BMD	6–9 years: daily 600 mg elemental calcium & 400 IU vitamin D3 10–14 years: daily 1350 mg elemental calcium & 460 IU vitamin D3	BMD, bone turnover markers, vitamin D level, and PTH	Vitamin D concentration significantly improved (p 0.001) Prevalence of low BMD significantly reduced by 56.29% (p < 0.05)	Low
Smuts et al. 2005 [2]	Double blinded, RCT 6 months	6–11 months	1,134	Residents in study location, not born prematurely or low birth weight, not severely wasted nor severely anaemic, no fever	WMM: weekly multiple micronutrient supplements (n = 283) DMM: daily multiple micronutrient supplement (n = 280) DI: daily iron supplement (n = 288) *Control group (P): placebo* *(n* = *283)*	CRP, Pb, retinol, Hb, and riboflavin level	The DMM group had a significantly greater weight gain, growing at an average rate of 207 g/mo compared with 192 g/mo for the WMM group, and 186 g/mo for the DI and P groups. DMM had significantly greater reduction in anaemia (-44% vs -35.1% and -29.9%), ID (-17.6% vs -13.7% and 9.3%) and VAD (-10.7% vs -4.3% and -11.4%) compared to DI and P groups (p < 0.05)	

*BMD* bone mineral density, *BMI* body mass index, *BMI-Z* body mass index for-age-Z score, *CRP* C-reactive protein, *HAZ* height-for-age-Z score, *Hb* haemoglobin, *IDA* iron deficiency anaemia, *LAZ* length-for-age-Z score, *LBW* low birth weight, *MMN* multi-micronutrient, *WAZ* weight-for-age-Z score, *WHZ* weight-for-height-Z score, *PF* plasma ferritin, *PTH* parathyroid hormone, *RCT* randomised controlled trial, *SF* serum ferritin, *sTfR* serum transferrin receptors

Most of the intervention studies (93%) demonstrated the effectiveness of the intervention in improving the children’s nutritional status, either through improved behaviours (e.g., optimal breastfeeding, exclusive breastfeeding for longer duration, better weaning practices, children consuming more food and more hygienic practices) in the nutrition sensitive interventions**,** or improved anthropometric indices and micronutrient biomarkers in the nutrition specific interventions (e.g., micronutrients supplementation and fortification). With respect to nutrition sensitive interventions, an evaluation study reported the success of the Government’s National Plan of Action for Nutrition (NPAN) and the National Targeted Programme on Hunger Eradication and Poverty Reduction [3] on improving child’s nutritional status through a variety of poverty reduction initiatives. Another three nutrition sensitive intervention studies using growth monitoring, positive deviance, and positive feeding practices approaches also reported significant reduction in the prevalence of severely malnourished [69], undernourished [71], and stunted children [70] through improved eating behaviours and feeding practices.

With respect to nutrition specific interventions, milk fortification improved height of infants aged 18–36 months by 1 cm on average (p < 0.01), weight by 0.43 kg (*p* < 0.01) and BMI-Z score by 0.015 (p < 0.05) after 5 months [86]. Similarly, children gained significantly more weight and height compared to the control group, after receiving fortified biscuits and milk which provided an extra 300 kcal/day [73]. Children of mothers who received multiple micronutrients supplementation during pregnancy were significantly taller than those receiving just iron and folic acid [77], however such effects were not found in Hanieh et al. 20I4 [74]. Infants were at a lower risk of anaemia if their mothers had taken the iron and folic acid supplement daily (OR = 0.31; 95% CI = 0.22–0.43), twice weekly (0.38; 95% CI = 0.26–0.54), or the multiple micronutrient (0.33; 95% CI = 0.23–0.48) during pregnancy compared to the placebo group [83]. Combined iron and zinc supplementations significantly improved the levels of haemoglobin (Hb), ferritin, and zinc (p < 0.0001) in children aged 4–7 months old after six months of intervention [72]. Similarly, prevalence of iron deficiency and anaemia was reduced in infants and children after receiving fortified foods for 5–6 months compared to the placebo group [78, 81, 82]. Supplementing children with 10mg of zinc daily resulted in weight gain by 0.5 kg on average (+ 0.1 kg, p < 0.001) and height by 1.5cm (+ 0.2 cm, *p* < 0.001) [80].

Only one uncontrolled trial investigated the effects of vitamin D and calcium supplementation [87], and the authors reported improved vitamin D concentrations (p < 0.001) and bone mineral density (BMD). Another study reported that after one month of intervention, both synthetic β-carotene supplementation (a precursor to vitamin A) and Momordica coincidences (gac) fruit showed significantly higher levels of β-carotene than in control group (p < 0.0001), but plasma retinol concentrations were significantly higher in children receiving gac fruit than those who received synthetic β-carotene (*p* < 0.01) [85].

## Discussion

### Prevalence of different forms of malnutrition in Vietnamese children

Our findings revealed that undernutrition (e.g., stunting) and MNDs (e.g., anaemia, iron, zinc and vitamin A deficiencies) were more prevalent in Vietnamese children aged < 5 years old compared to older children, whereas prevalence of overweight and obesity were rising in older children especially among primary school-aged children. Childhood undernutrition has gradually reduced over time in Vietnam [23]. This decrease in undernutrition aligns with the country’s Hunger Eradication and Poverty Reduction programme (HEPR) launched since 1992, which resulted in improved infrastructure in poor communities, greater financial support for ethnic poor families, and growth of the agriculture and aquaculture industries. In 1995, the NPAN indicated the government’s serious commitment to reducing malnutrition in the country [3]. These national programmes have shown positive outcomes on poverty reduction, subsequently resulting in improvement in child’s nutritional status and reduction in child undernutrition and MNDs [3].

**Fig. 4:**
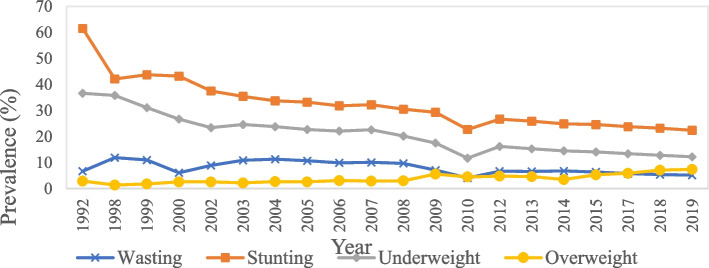
The prevalence (%) of wasting, stunting, underweight and overweight in Vietnamese children aged < 5 years old, from 1992 to 2019. Source: UNICEF/WHO/World Bank 2022

This finding was in line with the data reported by the UNICEF/WHO/World Bank Group [88] (Fig. 4), of a steady decline in wasting, stunting and underweight from 1998 to 2019, and a slow increase in overweight in children aged < 5 years old. Vietnam has made substantial progress towards achieving the targets set for the SDGs, which is to reduce the prevalence of stunting and wasting in children aged < 5 years old to below 20% and 5%, respectively [89]. However, ethnic minorities, especially those residing in poor and remote areas, are still being left behind, thus limiting the progress on national reductions [89]. This emphasises the urgent needs to focus prevention implementation on the most vulnerable populations, with strategies to address socioeconomic inequalities. On the other hand, Vietnam has failed to prevent the increase of childhood overweight and obesity. It is worth noting that for the first time in 2017, the prevalence of overweight in children aged < 5 years old overtook the prevalence of wasting in Vietnam. This has also been reflected in the increasing number of studies investigating childhood overweight and obesity since 2015–2022.

Compared to neighbouring SEA countries, prevalence of overweight children aged < 5 years old (3.5%) in Vietnam was lower than in Indonesia (11.5%) and Thailand (10.9%) in 2015 but higher than in Myanmar and Cambodia (< 2%) [90]. The prevalence of underweight in children aged 0.5–11 years old was lower in Vietnam (10.9% urban; 20.1% rural) compared to Indonesia (25.1% urban; 39.2% rural) but higher than Thailand (6.4% urban; 10.2% rural). Prevalence of anaemia was higher in Vietnam (22.5% urban; 27% rural) than Thailand (9.0% urban; 18.4% rural) and Indonesia (17.6% urban; 18.5% rural). Thus, the nutrition situation in Vietnam is not extreme for the region, with differences likely due to different economic and political circumstances, as well as different staple diets in each country.

### Determinants of childhood overweight and obesity

According to the ‘nutrition transition theory’ proposed by Popkin 1994 [91], rapid urbanisation and economic development, have contributed to the nutrition transition in Vietnam, from traditional diets rich in fresh fruits, vegetables and rice, to diets that are high in processed foods, refined grains, meat, dairy products, saturated fats, and sugars and are low in fibre [92, 93]. The price of food in Vietnam, especially high-sugar and processed foods decreased during the nutrition transition, making them increasingly accessible [94]. Unhealthier foods have become increasingly marketed towards children and accessible in outlets such as supermarkets, convenience shops and school cafeterias [7]. This has resulted in an increased risk of childhood overweight and obesity, and the development of double or triple burden of malnutrition.

Boys (*proximal factors*) are more likely to be overweight and obese compared to girls in Vietnam, especially in urban areas. Biological influences on diet (e.g., higher energy intake in boys), and sociocultural influences at individual, parental and familial level [95] are potential contributing factors. Girls are more likely to experience a higher-level of weight-related concerns, including desire to lose weight, compared to boys due to gender-based stereotypes [96]. Another reason could be due to male chauvinism, where parents regard boys as more valuable than girls [97], and parents may be more incline to overlook overweight and obesity status in sons compared to daughters [98]. In terms of *intermediate risk factors*, children living with their father only, had increased risk of being overweight and obese, compared to those with both their mother and father [39]. A possible explanation for this increased risk could be due to fathers being less likely to limit food access and monitor children’s food intake [99]. However, due to the low divorce rate in Vietnam, this may only represent 1.15% or even less of the population.

Children living in urban areas (*distal factors*) were more likely to be overweight and obese compared to rural areas [8]. This may be due to increased sedentary lifestyle, inactive transport methods to school (bus, car, motorbike), no recess activity due to fewer open spaces and playgrounds, more processed foods and fast food restaurants, and neighbourhoods that are less safe for walking and outdoor activities, alone [5, 19, 39, 55, 56, 63], which may have promoted weight gain in children. Although most studies in Vietnam reported that children living in wealthier families had a higher odd of obesity, but a recent study in Ho Chi Minh underscored that children living in lower-income families in urban areas are likely to have a higher odd of obesity than those living in higher-income families [7]. This may be attributed to various barriers to healthy eating and lifestyle unique to rural settings such as increased availability of low-cost energy-dense foods, inadequate grocery stores, lack of exercise facilities, and lack of nutritional knowledge [100, 101]. This trend may be similar in high-income countries.

### Determinants of child undernutrition and MNDs

It is well accepted that undernutrition and MNDs mainly results from inadequate intake of energy and nutrients, malabsorption, impaired metabolism, loss of nutrients, or increased nutritional requirements [102]. With respect to the *distal factors* of undernutrition, previous evidence reported that although much effort has been made to control child undernutrition and MNDs in Vietnam, prevalence still remains high, especially in ethnic minorities residing in poor and remote mountainous areas [65, 103]. Reasons may include food insecurities [104], poor diet quality, low socioeconomic status, low quality of health and nutrition service and high incidence of intestinal parasitic infections [21, 105].

With respect to environmental intermediate factors, improved household sanitation can reduce exposure to diarrhoeal diseases [48], reducing the risk of undernutrition and MNDs [106]. Household size was found to be positively associated with undernutrition [11, 23, 51], as children in large households may receive lower quantities of food, resulting in inadequate energy and nutrient consumption. In addition, preschool attendance, with three meals a day included, was found to be negatively associated with underweight and stunting [50]. Maternal factors, such as the mother’s height, BMI, and nutritional status were found to be positively associated with child growth indicators [107], emphasizing the need for pregnant women as well as women of reproductive age to have a healthy weight, or BMI, to increase the likelihood of producing healthy children. Parental mental disorders were also associated with child underweight and stunting [28, 46] which can be explained by the parent’s hindered ability to take care of their children and poor feeding practices [28].

With respect to proximal factors, children who were born with LBW were associated with increased risk of stunting, wasting, and underweight [11, 28]. LBW is a result of poor maternal nutritional status during pregnancy or adolescence, and preterm birth [108], and is commonly associated with reduced immune function and increased vulnerability to illness. Furthermore, our findings also emphasise the importance of optimal breastfeeding and complementary feeding practices in ensuring children receive adequate amount of nutrients for optimal growth, and to protect against infections [109]. Other studies reported that anaemia was mainly attributable to inadequate dietary iron intake, although malaria, deficiencies in other micronutrients such as vitamin A and selenium, being underweight, and age are also contributing factors [41, 65]. It is reported that young boys were at a higher risk of anaemia than girls [8]. This aligns with previous evidence that indicated that males are at a higher risk of iron deficiency compared to girls when aged < 10 years old [110]. It has been suggested that the hormonal effects on erythropoietin activity for red blood cell production, and higher pre- and postnatal growth rate in males (e.g., differences in body weight or proportion of lean and fat mass may affect iron metabolism) may have increased the susceptibility to anaemia in young boys [33, 61, 62]. Whereas in girls aged > 10 years old specifically, anaemia is likely attributable to regular blood loss through menstrual bleeding which depletes their iron stores [111]. Nonetheless, future investigations are needed to explore the effects of malaria and both nutritional and non-nutritional factors on anaemia.

Zinc deficiency varied by age, with a higher prevalence found in children aged 1–6 years old than those aged 6–11 months. Although the underlying reasons are yet to be explored, it is widely accepted that in low-and middle-income countries like Vietnam, the predominant rice-based diet with little animal produce, may result in insufficient dietary zinc intake to meet the high requirement needed for rapid growth at this age [84]. Serum zinc biomarker is known to be prone to over-estimation errors due to contamination at field collection and testing stage, which likely contributes to inaccurate assessment. However, the interpretation of the key determinants of MNDs is limited by the scarcity of MNDs data, primarily due to the limited investigation, laboratory facilities and testing capacity.

### Intervention and its effectiveness in addressing different forms of malnutrition

The majority of the intervention studies in Vietnam focus on addressing child undernutrition and MNDs in both rural and urban areas. Most studies investigated the impacts of daily multi-micronutrient supplementation (e.g., 13–15 micronutrients) or fortified foods (e.g., milk and biscuits) on children’s nutritional status. The dosage effects of supplements were investigated across different intervention studies, specifically in iron (10–60 mg), and folic acid (0.4–1.5 mg), whereas the dosage of zinc supplements given in the interventions were more consistent (10 mg). All supplementation interventions reported improvement in the child’s nutritional status in at least one of the outcomes.

Iron fortification has shown a positive effect on Hb, ferritin and body iron levels after 6 months of intervention in anaemic children from rural Vietnam who were not iron deficient [78]. This indicates that iron fortification may be effective in improving anaemia and iron status of anaemic children. The Government Decree 09/2016/ND-CP has mandated the fortification of salt with iodine, wheat flour with iron and zinc, and vegetable oil with vitamin A, which has been applied by many other countries, including developed countries. However, the implementation of Decree 09 still faces many difficulties in Vietnam. It is worth noting that two studies consistently reported negative interaction between zinc and iron co-supplementation on serum zinc levels, WAZ, or anaemia [72, 84]. Zinc supplementation alone was more effective at improving zinc deficiency and WAZ, whereas iron supplementation alone was more effective at reducing anaemia, compared to combined zinc and iron supplementation. The mechanism for this interaction between zinc and iron is likely due to the competitive absorption through a shared, non-specific pathway [112]. It is suggested that taking each supplement 30 min apart would be enough to limit their interactions and allow sufficient absorption of each micronutrient.

NPAN is considered one of the largest scaled national nutrition sensitive intervention programmes involving a variety of poverty reduction initiatives, and was found to be the most effective intervention in reducing child malnutrition [3]. Other nutrition sensitive intervention programmes included deworming, growth monitoring, nutritional rehabilitation, loan programmes, improved health care and positive deviance inquiry. Deworming showed no impact on Hb, ferritin and body iron status, and authors postulated that this may be due to the ‘light’ or ‘mild’ *Trichuris* infection among the children in this study population and hence showing an absence of effects. Thus, the effectiveness of deworming in improving Hb status in this population is still questionable and future research is warranted, particularly in areas where there are high rate of infection/inflammation [78]. Watanabe et al. 2005 reported a decrease in the prevalence of stunting following a nutrition intervention of growth monitoring and nutrition education [70], this intervention involved educating children and parents about locally available nutritious foods. Furthermore, positive deviance approaches and childcare practices which led to improved feeding practices and eating behaviours have resulted in improvement in anthropometric indices [69, 71].

### Current evidence gaps and future recommendations

Despite the large amount of data collected and analysed, there is limited evidence on the determinants, especially for MNDs and overnutrition. No intervention studies were found aimed at addressing childhood overweight and obesity in Vietnam, suggesting the need for more research to fill in evidence gaps on effective approaches to combat childhood overweight and obesity. Future intervention studies should focus on “double-duty actions” that aim to simultaneously address multiple forms of malnutrition. Particularly for intervention studies, it is essential to include randomisation and blinding procedures and withdrawal description (where applicable), to improve the validity and quality of the study outcomes.

The National Nutrition Strategy (NNS) 2021–2030, with a vision towards 2045 which was ratified by the Vietnamese Prime Minister (NNS Decision No. 02/QD-TTg) has also included indicators on overweight and obesity in children and adolescents, showing the Vietnamese government’s commitment in addressing the emerging issue of overweight and obesity [113]. Vietnamese government’s priorities align with the WHO plan for controlling the global obesity epidemic, which is to promote a whole-society and cross-sectoral approach, to address the obesogenic environment that is negatively influencing children’s health and nutrition [114]. However, more evidence on effective strategies is needed to better develop and implement such policies and intervention programmes, including introduction of a sugary sweetened beverages (SSB) tax, restricting the marketing and advertising of unhealthy foods and drinks, and front of pack nutrition labelling [113, 115]. Joint efforts from local government, schools, parents, and food industries will be required to ensure the availability and accessibility of more affordable and healthier options of food products to make them more appealing to consumers. Education programmes should be strengthened to generate public awareness on healthy eating and active lifestyle to promote healthy eating behaviour and lifestyle in children and parents/caregivers.

### Strengths and limitations

A key strength of this review is the comprehensive and systematic review of the existing evidence on all forms of childhood malnutrition (undernutrition, overnutrition and MNDs) in Vietnamese children. The findings from this review allow us to identify the shared determinants of different forms of malnutrition and the type of interventions available in this region, which is essential to develop effective double-duty actions to simultaneously address all forms of malnutrition. The use of the standard PRISMA method for a systematic review and a standardised search strategy means that this review can be reproduced and compared with other reviews following the same format and using the same search strategy. One of the limitations of this review is the heterogeneous nature of the study outcomes, limiting the possibility to perform a meta-analysis on the extracted data. Lack of available data prevents reliable conclusions to be drawn, especially regarding MNDs and overnutrition, thus future research is warranted. Only studies reported in English were included, and this may have limited the scope.

## Conclusion

Undernutrition is still affecting one quarter of Vietnamese children aged under 5 despite a significant reduction over the last decade. MNDs remain a serious problem, particularly deficiencies in iron, zinc, and vitamin D, whilst childhood overweight and obesity are rapidly rising in school aged children, indicating the existence of the triple burden of malnutrition in this region. This finding highlights the needs for double duty actions, and these actions should be tailored based on regional-level needs, with strategies to address demographic and socioeconomic disparities to simultaneously and effectively address different forms of child malnutrition in Vietnam. Most of the nutrition-specific intervention strategies including supplementation and food fortification demonstrated positive outcomes; either through improvements in anthropometric indices, micronutrient biomarkers, or diet-related behaviours. Large-scale, nationwide nutrition-sensitive intervention programmes that collectively address different environmental influences on children’s health and food choices such as food availability, education, health care, and access to water and sanitation are more likely to be effective in promoting optimal child growth in the long term. However, evidence on the key determinants and effective intervention strategies, especially on MNDs and childhood overweight and obesity are still too limited to adequately inform policy decision, thus future research is warranted.

### Supplementary Information

**Supplementary Material 1.**

## Data Availability

The datasets used and/or analysed during the current study are available from the corresponding author on reasonable request.

## Abbreviations

BMD: Bone mineral density
BMI: Body mass index
BMI-Z: Body mass index for-age-Z score
CI: Confidence interval
CRP: C-reactive protein
HAZ: Height-for-age-Z score
Hb: Haemoglobin
IDA: Iron deficiency anaemia
IOTF: International obesity task force
LAZ: Length-for-age-Z score
LBW: Low birth weight
LMAC: Left mid arm circumference
MMN: Multi-micronutrient
MNDs: Micronutrient deficiencies
MUAC: Mid upper arm circumference
ND: No data
OR: Odds ratio
PF: Plasma ferritin
PTH: Parathyroid hormone
SD: Standard deviation
SDGs: Sustainable development goals
SF: Serum ferritin
SSF: Skinfold thickness
SSSF: Subscapular skinfold thickness
sTfR: Serum transferrin receptors
TSFT: Tricep skinfold thickness
WAZ: Weight-for-age-Z score
WHO: World Health Organisation
WHZ: Weight-for-height-Z score
WLZ: Weight-for-length-Z score
25(OH)D: 25-hydroxy vitamin D
