# Supplementary material for: Prevalence, determinants, intervention strategies and current gaps in addressing childhood malnutrition in Vietnam: a systematic review
Source: BMC Public Health. 2024 Apr 4;24:960. doi: 10.1186/s12889-024-18419-8 (PMC10996139; doi:10.1186/s12889-024-18419-8)
Supplement: Supplementary file 1 — Supplementary Material 1. [file 12889_2024_18419_MOESM1_ESM.docx]

**Title: Prevalence, determinants, intervention strategies and current gaps in addressing childhood malnutrition in Vietnam: a systematic review**

### Charlotte Mondon^1^, Pui Yee Tan^1*^, Chong Ling Chan^1^, Thuy Tran Nga ^2^, Yun Yun Gong^1*^

**Supplementary Table 1 (Table S1): Search strategy used in each database**

1. **PubMed**

| **No** | **Topic** | **Search terms** | **Results** |
| --- | --- | --- | --- |
| #1 | Malnutrition indicators | "Child Nutrition Disorders"[Mesh] OR "malnutrition"[MeSH] OR "Overnutrition"[MeSH] | 38686 |
| #2 |  | malnutrition[tiab] OR malnourish*[tiab] OR overnutrition[tiab] OR over-nutrition[tiab] OR overnourish*[tiab] OR over-nourish*[tiab] OR obese[tiab] OR obesity[tiab] OR overweight[tiab] OR over-weight[tiab] OR underweight[tiab] OR under-weight[tiab] OR undernourish*[tiab] OR under-nourish*[tiab] OR undernutrition[tiab] OR under-nutrition[tiab] OR stunted[tiab] OR stunting[tiab] OR wasted[tiab] OR wasting[tiab] OR thinness[tiab] | 463,217 |
| #3 | Population (children) | “child”[MeSH] OR “child, preschool” [MeSH] OR “adolescent”[MeSH] | 3,275,642 |
| #4 |  | child*[tiab] OR children[tiab] OR preschool[tiab] OR pre-school[tiab] OR infant[tiab] OR infancy[tiab] OR adolescen*[tiab] OR "school child"[tiab] OR "school children"[tiab] | 1,955,587 |
| #5 | Country (Vietnam) | "Vietnam"[MeSH] | 14,092 |
| #6 |  | vietnam[tiab] OR vietnamese[tiab] | 21,413 |
| #7 | Prevalence and determinants | "Prevalence"[Mesh] OR "Risk Factors"[Mesh] | 1,166,918 |
| #8 |  | determinant*[tiab] OR predictor*[tiab] OR indicator*[tiab] OR prevalence[tiab] OR factor*[tiab] OR driver*[tiab] OR barrier*[tiab] OR cause*[tiab] OR influencer*[tiab] | 7,362,391 |
| #9 | Micronutrient deficiencies | deficien*[tiab] OR inadequa*[tiab] OR insufficien*[tiab] OR low[tiab] OR lower[tiab] OR reduce*[tiab] | 7,082,876 |
| #10 |  | micronutrient*[tiab] OR iron[tiab] OR ferritin OR transferrin[tiab] OR retinol[tiab] OR zinc[tiab] OR folate[tiab] OR "folic acid*"[tiab] OR anaemia[tiab] OR anemia[tiab] OR iodine[tiab] OR "vitamin A"[tiab] OR "vitamin B9"[tiab] | 636,798 |
| #11 |  | #9 and #10 | 238,527 |
| #12 |  | "Anemia, Iron-Deficiency"[Mesh] OR "Vitamin A Deficiency"[Mesh] OR "Folic Acid Deficiency"[Mesh] | 21,838 |
| #13 | Interventions | "Breast Feeding"[Mesh] OR "Biofortification"[Mesh] OR "Food Security"[Mesh] OR "Food Safety"[Mesh] OR "Family Planning Services"[Mesh] OR "Maternal Health"[Mesh] OR "Maternal Health Services"[Mesh] | 204,858 |
| #14 |  | "nutrition specific"[tiab] OR "nutrition-specific"[tiab] OR "nutrition sensitive"[tiab] OR "nutrition-sensitive"[tiab] | 445 |
| #15 |  | (diet[tiab] OR dietary[tiab] OR food[tiab]) | 955,738 |
| #16 |  | (fortifi*[tiab] OR diversif*[tiab]) | 57,371 |
| #17 |  | #15 AND #16 | 9,092 |
| #18 |  | breastfeeding[tiab] OR breastfed[tiab] OR "complementary feeding"[tiab] OR "complementary food"[tiab] OR "cash transfer"[tiab] OR "cash-transfer"[tiab] OR "school feeding"[tiab] OR "school meal"[tiab] OR "therapeutic food"[tiab] OR "ready-to-use therapeutic food"[tiab] | 47,027 |
| #19 |  | "food security"[tiab] OR "food safety"[tiab] OR "safety net*"[tiab] OR "maternal health"[tiab] OR "women's empowerment"[tiab] OR "female empowerment" [tiab] OR "gender equality"[tiab] OR education*[tiab] OR "water, sanitation and hygiene"[tiab] OR "water, sanitation"[tiab] OR "water and sanitation"[tiab] OR "family planning"[tiab] OR supplement*[tiab] OR 'child protection'[tiab] OR "health service*"[tiab] | 1,243,455 |
| #20 |  | intervention*[tiab] OR programme*[tiab] OR program*[tiab] OR prevention*[tiab] | 2,558,349 |
| #21 | Combined search | #1 OR #2 | 618433 |
| #22 |  | #3 OR #4 | 3,944,059 |
| #23 |  | #5 OR #6 | 24,056 |
| #24 |  | #7 OR #8 | 7,754,548 |
| #25 |  | #11 OR #12 | 2439943 |
| #26 |  | #13 OR #14 OR #17 OR #18 OR #19 OR #20 | 3575125 |
| #30 |  | (#21 OR #24) AND #22 AND #23 AND (#24 OR #25) | **620** |

1. **Embase**

| **No** | **Topic** | **Search terms** | **Results** |
| --- | --- | --- | --- |
| #1 | Malnutrition indicators | "Nutritional Disorder"/ OR "malnutrition"/ OR "Overnutrition"/ | 72,561 |
| #2 |  | (malnutrition or malnourish* or overnutrition or over-nutrition or overnourish* or over-nourish* or obese or obesity or overweight or over-weight or underweight or under-weight or undernourish* or under-nourish* or undernutrition or under-nutrition or stunted or stunting or wasted or wasting or thinness).ab,kw,ti. | 616,617 |
|  |  |  |  |
|  |  |  |  |
|  |  |  |  |
| #3 | Population (children) | “child”/ OR “child, preschool” / OR “adolescent”/ | 2,349,274 |
| #4 |  | (child* or children or preschool or pre-school or infant or infancy or adolescen* or "school child" or "school children").ab,kw,ti. | 1,956,537 |
| #5 | Country (Vietnam) | "Viet nam"/ | 16,544 |
| #6 |  | (Vietnam or Vietnamese).ab,kw,ti. | 22,065 |
| #7 | Prevalence and determinants | "Prevalence"/ OR "Risk Factors"/ | 1,823,329 |
| #8 |  | (determinant* or predictor* or indicator* or prevalence or factor* or driver* or barrier* or cause* or influencer*).ab,kw,ti. | 8,343,490 |
| #9 | Micronutrient deficiencies | (deficien* or inadequa* or insufficien* or low or lower or reduce*).ab,kw,ti. | 7,978,144 |
| #10 |  | (micronutrient* or iron or ferritin or transferrin or retinol or zinc or folate or "folic acid*" or anaemia or anemia or iodine or "vitamin A" or "vitamin B9").ab,kw,ti. | 624,108 |
| #11 |  | #9 AND #10 | 277,547 |
| #12 |  | "Anemia, Iron-Deficiency"/ OR "retinol Deficiency"/ OR "Folic Acid Deficiency"/ | 34,406 |
| #13 | Interventions | "Breast Feeding"/ OR "Biofortification"/ OR "Food Security"/ OR "Food Safety"/ OR "Family Planning"/ OR "Maternal Health"/ | 110,460 |
| #14 |  | ("nutrition specific" or "nutrition-specific" or "nutrition sensitive" or "nutrition-sensitive").ab,kw,ti. | 516 |
| #15 |  | (diet or dietary or food).ab,kw,ti. | 1,001,578 |
| #16 |  | (fortifi* or diversif*).ab,kw,ti. | 57,833 |
| #17 |  | #15 AND #16 | 9,826 |
| #18 |  | (breastfeeding or breastfed or "complementary feeding" or "complementary food" or "cash transfer" or "cash-transfer" or "cash-based transfer" or "school feeding" or "school meal" or "therapeutic food" or "ready-to-use therapeutic food").ab,kw,ti. | 44,828 |
| #19 |  | ("food security" or "food safety" or "safety net*" or "maternal health" or "women's empowerment" or "women* empowerment" or "female empowerment" or "gender equality" or education* or "water, sanitation and hygiene" or "water, sanitation" or "water and sanitation" or "family planning" or supplement* or 'child protection' or "maternal mental heath" or "health service*").ab,kw,ti. | 1,355,026 |
| #20 |  | (intervention* or programme* or program* or prevention*).ab,kw,ti. | 3,069,727 |
| #21 | Combined search | #1 OR #2 | 639,362 |
| #22 |  | #3 OR #4 | 2,993,470 |
| #23 |  | #5 OR #6 | 24,461 |
| #24 |  | #7 OR #8 | 8,781,840 |
| #25 |  | #11 OR #12 | 288,622 |
| #26 |  | #13 OR #17 OR #18 OR #19 OR #20 | 4,039,259 |
| #27 |  | (#21 OR #25) AND #22 AND #23 AND (#24 OR #26) | **722** |

1. **Scopus**

| **No** | **Topic** | **Search terms** | **Results** |
| --- | --- | --- | --- |
| #1 | Malnutrition indicators | (TITLE-ABS-KEY ( malnutrition ) OR TITLE-ABS-KEY ( malnourish* ) OR TITLE-ABS-KEY ( malnourishment ) OR TITLE-ABS-KEY ( under?nutrition ) OR TITLE-ABS-KEY ( under?nourish* ) OR TITLE-ABS-KEY ( stuting ) OR TITLE-ABS-KEY ( stunted ) OR TITLE-ABS-KEY ( over?nutrition ) OR TITLE-ABS-KEY ( over?nourish* ) OR TITLE-ABS-KEY ( wasting ) OR TITLE-ABS-KEY ( wasted ) OR TITLE-ABS-KEY ( thinness ) OR TITLE-ABS-KEY ( underweight ) OR TITLE-ABS-KEY ( overweight ) OR TITLE-ABS-KEY ( obesity ) OR TITLE-ABS-KEY ( obese)) | 734,462 |
| #2 | Population (children) | TITLE-ABS-KEY ( child ) OR TITLE-ABS-KEY ( children ) OR TITLE-ABS-KEY ( preschool ) OR TITLE-ABS-KEY ( pre-school ) OR TITLE-ABS-KEY ( infant ) OR TITLE-ABS-KEY ( infancy ) OR TITLE-ABS-KEY ( adolescen* ) OR TITLE-ABS-KEY ( "SCHOOL CHILD*" ) | 5,258,352 |
| #3 | Country (Vietnam) | TITLE-ABS-KEY ( vietnam ) OR TITLE-ABS-KEY ( vietnamese ) | 69,747 |
| #4 | Prevalence and determinants | ( TITLE-ABS-KEY ( determinant? ) OR TITLE-ABS-KEY ( predictor? ) OR TITLE-ABS-KEY ( indicator? ) OR TITLE-ABS-KEY ( factor? ) OR TITLE-ABS-KEY ( driver? ) OR TITLE-ABS-KEY ( barrier? ) OR TITLE-ABS-KEY ( cause? ) OR TITLE-ABS-KEY ( influence? ) ) | 12,224,605 |
| #5 | Micronutrient deficiencies | ( TITLE-ABS-KEY ( deficien* ) OR TITLE-ABS-KEY ( inadequa* ) OR TITLE-ABS-KEY ( insufficien* ) OR TITLE-ABS-KEY ( low ) OR TITLE-ABS-KEY ( lower ) OR TITLE-ABS-KEY ( reduce* ) ) | 17,754,016 |
| #6 |  | ( TITLE-ABS-KEY ( micronutrient? ) OR TITLE-ABS-KEY ( "vitamin a" ) OR TITLE-ABS-KEY ( "vitamin b9" ) OR TITLE-ABS-KEY ( iron ) OR TITLE-ABS-KEY ( ferritin ) OR TITLE-ABS-KEY ( transferrin ) OR TITLE-ABS-KEY ( retinol ) OR TITLE-ABS-KEY ( zinc ) OR TITLE-ABS-KEY ( folate ) OR TITLE-ABS-KEY ( "folic acid" ) OR TITLE-ABS-KEY ( an?emi* ) OR TITLE-ABS-KEY ( iodine ) ) | 1,999,297 |
| #7 |  | #5 AND #6 | 688,045 |
| #8 | Interventions | TITLE-ABS-KEY ( "nutrition specific" ) OR TITLE-ABS-KEY ( "nutrition-specific" ) OR TITLE-ABS-KEY ( "nutrition sensitive" ) OR TITLE-ABS-KEY ( "nutrition-sensitive" ) | 693 |
| #9 |  | TITLE-ABS-KEY ( diet ) OR TITLE-ABS-KEY ( food ) OR TITLE-ABS-KEY ( dietary ) | 2,413,412 |
| #10 |  | TITLE-ABS-KEY ( fortif* ) OR TITLE-ABS-KEY ( diversif* ) | 185,609 |
| #11 |  | #9 AND #10 | 30,698 |
| #12 |  | TITLE-ABS-KEY ( breastfeeding ) OR TITLE-ABS-KEY ( breastfed ) OR TITLE-ABS-KEY ( "complementary food" ) OR TITLE-ABS-KEY ( "complementary feeding" ) OR TITLE-ABS-KEY ( "cash transfer" ) OR TITLE-ABS-KEY ( "cash-transfer" ) OR TITLE-ABS-KEY ( "cash-based transfer" ) OR TITLE-ABS-KEY ( "school feeding" ) OR TITLE-ABS-KEY ( "school meal" ) OR TITLE-ABS-KEY ( "therapeutic food" ) OR TITLE-ABS-KEY ( "ready-to-use therapeutic food" ) | 49,597 |
| #13 |  | TITLE-ABS-KEY ( "food security" ) OR TITLE-ABS-KEY ( "food safety" ) OR TITLE-ABS-KEY ( "safety net*" ) OR TITLE-ABS-KEY ( "maternal health" ) OR TITLE-ABS-KEY ( "women* empowerment" ) OR TITLE-ABS-KEY ( "female empowerment" ) OR TITLE-ABS-KEY ( "gender equality" ) OR TITLE-ABS-KEY ( "education*" ) OR TITLE-ABS-KEY ( "water, sanitation and hygiene" ) OR TITLE-ABS-KEY ( "water, sanitation" ) OR TITLE-ABS-KEY ( "water and sanitation" ) OR TITLE-ABS-KEY ( "family planning" ) OR TITLE-ABS-KEY ( "supplement*" ) OR TITLE-ABS-KEY ( "child protection" ) OR TITLE-ABS-KEY ( "maternal mental health" ) OR TITLE-ABS-KEY ( "health service*" ) | 4,179,043 |
| #14 |  | TITLE-ABS-KEY ( intervention* ) OR TITLE-ABS-KEY ( programme* ) OR TITLE-ABS-KEY ( program* ) OR TITLE-ABS-KEY ( prevention* ) | 6,433,450 |
| #15 |  | #8 or #11 or #12 or #13 or #14 | 9,543,296 |
| #16 | Combined search | (#1 OR #7) AND #2 AND #3 AND (#4 OR #15) | **571** |

1. **Web of Science**

| **No** | | | **Topic** |
| --- | --- | --- | --- |
| #1 | Malnutrition indicators | **Search terms** | **Results** |
| #2 | Population (children) | (((((((((((((((TS=(malnutrition)) OR TS=(malnourish*)) OR TS=(over?nutrition)) OR TS=(over?nourish*)) OR TS=(overnourish*)) OR TS=(over-nourish*)) OR TS=(obese)) OR TS=(over?weight)) OR TS=(under?weight)) OR TS=(under?nourish*)) OR TS=(under?nutrition)) OR TS=(stunting)) OR TS=(stunted)) OR TS=(wasting)) OR TS=(wasted)) OR TS=(thinness) | 803,861 |
| #3 | Country (Vietnam) | (((((((TS=(child*)) OR TS=(children)) OR TS=(pre?school)) OR TS=(infant?)) OR TS=(infancy)) OR TS=(adolescen*)) OR TS=("school child")) OR TS=( "school children") | 2,612,330 |
| #4 | Prevalence and determinants | ((TS=(Vietnam)) OR TS=(Vietnamese)) | 62,701 |
| #5 | Micronutrient deficiencies | (((((((TS=(prevalence)) OR TS=(predictor?)) OR TS=(indicator?)) OR TS=(determinant?)) OR TS=(factor?)) OR TS=(driver?)) OR TS=(barrier?)) OR TS=(cause?) | 7,757,209 |
| #6 | Micronutrient deficiencies  Interventions | (((((TS=(deficien*)) OR TS=(inadequa*)) OR TS=(insufficien*)) OR TS=(low)) OR TS=(lower)) OR TS=(reduce*) | 13,062,282 |
| #7 |  | (((((((((((TS=(micronutrient?)) OR TS=(iron)) OR TS=(ferritin)) OR TS=(transferrin)) OR TS=(retinol)) OR TS=(zinc)) OR TS=(folate)) OR TS=("folic acid*")) OR TS=(an?emi*)) OR TS=(iodine)) OR TS=("vitamin A")) OR TS=("vitamin B9") | 1,286,517 |
| #8 |  | #5 AND #6 | 428,282 |
| #9 | Interventions  Combined search | (((TS=("nutrition specific")) OR TS=("nutrition-specific")) AND TS=("nutrition sensitive")) OR TS=("nutrition-sensitive") | 510 |
| #10 |  | (((TS=(diet)) OR TS=(dietary)) OR TS=(food)) | 1,664,589 |
| #11 |  | ((TS=(fortif*)) OR TS=(diversif*)) | 155,528 |
| #12 |  | #9 AND 10 | 20,034 |
| #13 |  | ((((((((((TS=(breastfeeding)) OR TS=(breastfed)) OR TS=("complementary food")) OR TS=("complementary feeding")) OR TS=("cash transfer")) OR TS=("cash-transfer")) OR TS=("cash-based transfer")) OR TS=("school feeding")) OR TS=("school meal")) OR TS=("therapeutic food")) OR TS=("ready-to-use therapeutic food") | 38,491 |
| #14 |  | (((((((((((((((TS=("food security")) OR TS=("food safety")) OR TS=("safety net*")) OR TS=("maternal health")) OR TS=("women* empowerment" )) OR TS=("female empowerment")) OR TS=("gender equality")) OR TS=("education*")) OR TS=("water, sanitation OR hygiene")) OR TS=("water, sanitation")) OR TS=("water OR sanitation")) OR TS=("family planning")) OR TS=("supplement*")) OR TS=("child protection")) OR TS=("maternal mental health")) OR TS=("health service*") | 2,215,903 |
| #15 |  | (((TS=(intervention*)) OR TS=(programme*)) OR TS=(program*)) OR TS=(prevention*) | 4,065,638 |
| #16 |  | #8 OR #11 OR #12 OR #13 OR #14 | 5,756,744 |
|  |  |  |  |
